# Developing evidence-based recommendations for optimal interpregnancy intervals in high-income countries: protocol for an international cohort study

**DOI:** 10.1136/bmjopen-2018-027941

**Published:** 2019-01-29

**Authors:** M Luke Marinovich, Annette K Regan, Mika Gissler, Maria C Magnus, Siri Eldevik Håberg, Amy M Padula, Jonathan A Mayo, Gary M Shaw, Stephen Ball, Eva Malacova, Amanuel T Gebremedhin, Natasha Nassar, Cicely Marston, Nick de Klerk, Ana Pilar Betran, Gavin F Pereira

**Affiliations:** 1 School of Public Health, Curtin University, Bentley, Western Australia, Australia; 2 School of Public Health, Texas A and M University, College Station, Texas, USA; 3 Information Services Department, National Institute for Health and Welfare, Helsinki, Finland; 4 Department of Neurobiology, Care Sciences and Society, Karolinska Institute, Stockholm, Sweden; 5 MRC Integrative Epidemiology Unit, University of Bristol, Bristol, UK; 6 Population Health Sciences, Bristol Medical School, Bristol, UK; 7 Centre for Fertility and Health (CeFH), Norwegian Institute of Public Health, Oslo, Norway; 8 Department of Obstetrics, Gynecology and Reproductive Sciences, University of California, San Francisco, California, USA; 9 Department of Pediatrics, Stanford University, Stanford, California, USA; 10 School of Nursing, Midwifery and Paramedicine, Curtin University, Perth, Western Australia, Australia; 11 Menzies Centre for Health Policy, School of Public Health, University of Sydney, Sydney, New South Wales, Australia; 12 Faculty of Public Health and Policy, London School of Hygiene and Tropical Medicine, London, UK; 13 Telethon Kids Institute, University of Western Australia, Subiaco, Western Australia, Australia; 14 UNDP/UNFPA/UNICEF/WHO/World Bank Special Programme of Research, Development and Research Training in Human Reproduction, Department of Reproductive Health and Research, World Health Organization, Geneva, Switzerland

**Keywords:** pregnancy, birth intervals, family planning, siblings, preterm birth, fetal growth restriction

## Abstract

**Introduction:**

Short interpregnancy interval (IPI) has been linked to adverse pregnancy outcomes. WHO recommends waiting at least 2 years after a live birth and 6 months after miscarriage or induced termination before conception of another pregnancy. The evidence underpinning these recommendations largely relies on data from low/middle-income countries. Furthermore, recent epidemiological investigations have suggested that these studies may overestimate the effects of IPI due to residual confounding. Future investigations of IPI effects in high-income countries drawing from large, population-based data sources are needed to inform IPI recommendations. We aim to assess the impact of IPIs on maternal and child health outcomes in high-income countries.

**Methods and analysis:**

This international longitudinal retrospective cohort study will include more than 18 million pregnancies, making it the largest study to investigate IPI in high-income countries. Population-based data from Australia, Finland, Norway and USA will be used. Birth records in each country will be used to identify consecutive pregnancies. Exact dates of birth and clinical best estimates of gestational length will be used to estimate IPI. Administrative birth and health data sources with >99% coverage in each country will be used to identify maternal sociodemographics, pregnancy complications, details of labour and delivery, birth and child health information. We will use matched and unmatched regression models to investigate the impact of IPI on maternal and infant outcomes, and conduct meta-analysis to pool results across countries.

**Ethics and dissemination:**

Ethics boards at participating sites approved this research (approval was not required in Finland). Findings will be published in peer-reviewed journals and presented at international conferences, and will inform recommendations for optimal IPI in high-income countries. Findings will provide important information for women and families planning future pregnancies and for clinicians providing prenatal care and giving guidance on family planning.

**Strengths and limitations of this study**

- With data from four countries and over 18 million pregnancies, this will be the largest cohort study to investigate IPI in high-income countries.
- The size of this cohort will allow strict control for confounding through matching multiple pregnancies within women, and permit the investigation of IPI effects among subpopulations.
- Collaboration between countries and the use of individual-level data will minimise methodological heterogeneity.
- Data items such as socioeconomic status vary between countries in their completeness and their method of measurement, limiting the extent to which such variables may be incorporated as covariates.
- Data on early pregnancy loss are poorly or inconsistently captured in routine data collection, which may result in misclassification of IPI.

## Introduction

Interpregnancy interval (IPI), or the time from birth to conception of the next pregnancy, has been identified as a potentially modifiable risk factor linked to adverse perinatal outcomes. Currently, WHO recommends that women wait at least 2 years after a live birth and 6 months after early pregnancy loss before conceiving again to reduce the risk of adverse maternal and perinatal outcomes.^1^ This recommendation is based on observational studies, mostly from low/middle-income countries, that have demonstrated associations between short IPI and adverse pregnancy outcomes,^2^ most notably maternal mortality,^3^ small for gestational age (SGA), term low birth weight (LBW),^4^ preterm prelabour rupture of membranes,^5 6^ preterm birth (PTB)^4 7 8^ and birth defects.^9 10^ Furthermore, long IPI has been shown to be associated with higher risk of outcomes such as fetal death,^11^ LBW, PTB, SGA^12^ and pre-eclampsia.^11 13^

Several theories have been proposed to explain these associations. Short IPIs may leave insufficient time to recover from maternal nutrient deficits, which can lead to fetal–maternal competition for essential nutrients.^14^ This can be exacerbated by lactation^15^ and malnourishment, in both high-income and low-income countries.^16 17^ Short IPIs might also leave insufficient recovery time from inflammatory processes from the previous pregnancy that extend into the next pregnancy.^2^ Long IPIs may result in the loss of adaptive benefits to the mother from a previous birth, resulting in a return to a state equivalent to primigravida.^17^ An alternative hypothesis is that the observed associations between IPI and adverse pregnancy outcomes can be explained, at least partially, by systematic bias.^18^ A spurious association would result if time to conception were independently associated with other factors causally linked to adverse pregnancy outcomes in the subsequent pregnancy.^19 20^ For example, patterns in IPI are associated with maternal age, socioeconomic status (SES), breastfeeding and other antenatal, postnatal or postpartum practices.^21 22^ Furthermore, many factors that can confound the associations between IPI and adverse pregnancy outcomes, such as psychosocial determinants of health, are difficult to measure. Undoubtedly, the greatest challenge for observational studies is to comprehensively account for such confounding factors. High quality studies have adjusted for potential confounders, but statistical adjustment is rarely complete. Residual unmeasured confounding can lead to bias^23^ of the observed effect of IPI on adverse pregnancy outcomes. Importantly, as the mechanisms by which IPI affects specific pregnancy outcomes are not well understood, direct adjustment cannot be made for confounders that remain unknown. Under this scenario, the current state of knowledge on the effects of IPI is clinically unreliable.

**Figure 1:**
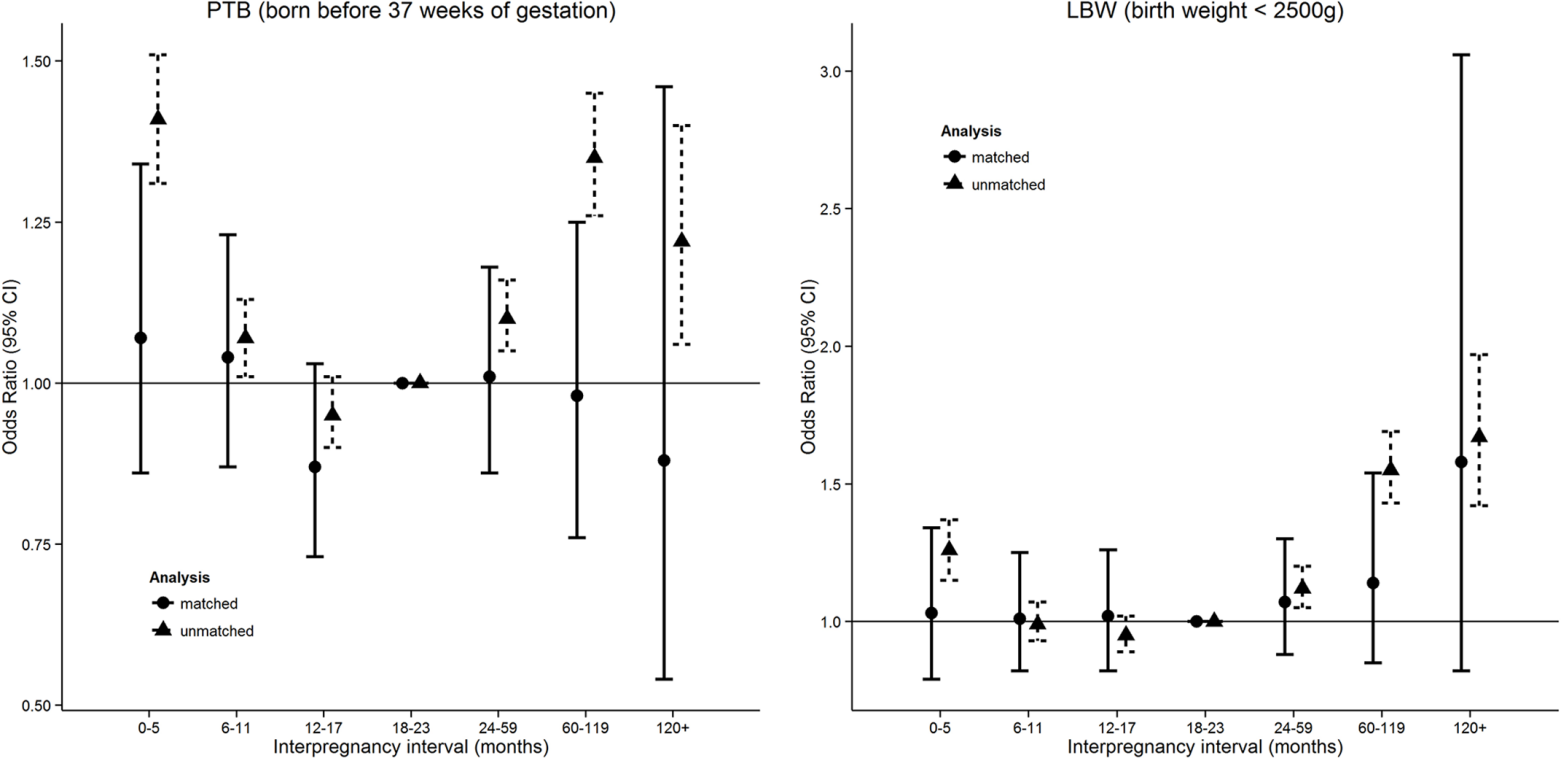
Effects of interpregnancy interval on preterm birth (PTB) and low birth weight (LBW), with and without matching pregnancies to the same women, Western Australia, 1980–2010. Figures produced from statistics reported in Ball et al. 2014^26^.

In recent years, studies have attempted to address the issue of bias from unmeasured confounding through a matched longitudinal study design.^24–26^ Longitudinal study designs which match pregnancies to the same women^26 27^ can better overcome confounding introduced by unknown, unmeasured and inaccurately measured factors that tend to vary between mothers but remain similar between pregnancies.^24 25 28^ Findings from these studies suggest the effects of IPI reported from unmatched models are overestimated. Considerable reductions in the U-shape effect of IPI are observed after matching pregnancies to the same women (figure 1),^26^ indicating support for the systematic bias hypothesis. These results provide a persuasive argument that some of the IPI effect observed in past studies may be attributable to confounders that vary between women (eg, heritable/genetic, socioeconomic, recurrent health-related behaviour, unknown/unmeasured), but can be effectively controlled under a longitudinal matched design.

Although these results cast doubt over the adverse effects of IPI, the existence of adverse associations with perinatal outcomes and the optimal IPI period remains unclear due to smaller sample sizes for matched analyses. CIs around estimates are typically wider for matched analyses which rely on women having at least two IPIs during the study period, particularly for the effects of longer IPI (>60 months). In addition, the selection of women with more than two pregnancies in matched analyses limits generalisability to all women. Several other gaps remain in our knowledge around IPI effects (table 1). First, the majority of studies have so far been restricted to low-income and middle-income countries. For example, the current WHO recommendation for IPI following early pregnancy loss are based on the findings from a single, albeit large, study in Latin America,^4^ and it remains unclear as to whether the findings are relevant to high-income countries. Second, some of the most pertinent questions for women in high-income countries, such as those related to obstetric context, have not yet been addressed. Additional data from high-income countries and more robust results are needed.

**Table 1:** Significant gaps in knowledge needed to inform recommendations for optimal interpregnancy interval (IPI) recommendations in high-income countries

Knowledge gap	Description
What are the IPI effects after eliminating confounding from between-women comparisons?	Few past studies have addressed confounding by matching pregnancies to the same women.^1^
Can IPI effects be observed later in childhood?	No past studies have investigated outcomes beyond the neonatal period, such as hospitalisation in early childhood.
What are the optimal IPIs for which risks are minimised?	Although knowledge of harmful IPIs is important, health is optimised by identification of optimal IPIs for which risks are minimised.^1^
What are the IPI effects for a wide range of outcomes of both the mother and child?	The vast majority of studies to date investigate a single endpoint for a single group (mother or child). IPI recommendations require a wide range of health endpoints for both mother and child.
What are the context-specific IPI recommendations?	Guideline IPIs are needed for subpopulations, for example, for women who attempt labour after caesarean birth to avoid uterine rupture,^29^ and for women after stillbirth due to elevated risk of recurrence or faster maternal recovery. For women at advanced maternal age, delaying child birth even further due to IPI recommendations comes at the cost of competing risks of age-related morbidities.^49^

### Study aims

The primary aim of this study is to evaluate the impact of IPI on pregnancy outcomes in high-income countries. Secondary aims are to:

1. Evaluate the impact of IPI based on obstetric context: IPI consequences may differ for women based on obstetric history. For example, optimal IPI may vary for women who previously gave birth by caesarean section due to risk of uterine rupture after insufficient time for the uterine scar to heal.^29^ The effect of IPI may also vary depending on gestational age of the previous birth, since longer gestation could be expected to prolong the maternal nutrient deficits and inflammatory processes implicated in poorer outcomes for the subsequent birth, particularly with short IPI. Furthermore, recommendations of IPI after stillbirth require specific attention.^30^ Pregnancy intervals for women following a stillbirth or neonatal loss are approximately 1 year shorter than intervals for women without a loss.^31 32^ It has been speculated that this is due to parents wishing to counter the emotional loss of their infant or to minimise the delay in having a baby.^33^ However, a survey of obstetricians regarding the timing of pregnancy following pregnancy loss found approximately two-thirds of obstetricians endorse women waiting less than 6 months after stillbirth before trying to conceive a new pregnancy.^34^ This is incongruous with WHO IPI recommendations that women wait at least 6 months after pregnancy loss.
2. Evaluate the impact of IPI based on sociodemographic context: The IPI recommendations generated by this study may be most beneficial for those populations for whom we know short IPI and high parity (large family size) is prevalent. Within high-income countries, both family size and the IPI distribution can vary markedly according to race/ethnicity and SES.^35 36^ In this study, we will investigate the effects of IPI among women from different socioeconomic and racial/ethnic groups in multiple high-income countries.
3. Evaluate the potential influence of maternal age on the effect of IPI on pregnancy outcomes: In high-income countries, age at childbirth overall is increasing, with later age at first birth.^37–39^ Systematic reviews conclude that increased maternal age is associated with greater risk of a wide range of adverse obstetric outcomes,^40^ including perinatal mortality,^41^ PTB and LBW^42^ and pregnancy intervention, particularly caesarean section.^43^ In this study, we will test the hypothesis that there is an interaction between increased maternal age and IPI on the risk of adverse pregnancy outcomes. For each year of maternal age (ie, maternal age at first observed birth in the study period) and for different IPIs, we will calculate the risk of adverse pregnancy outcomes. This will provide valuable information on any combined risks of maternal age and IPI for older women conceiving their second (or later) child.

## Methods and analysis

### Study design and population

We will conduct a longitudinal retrospective cohort study on the effects of IPI on maternal and child health outcomes using individual-level records with near complete coverage of all births (>99%) in Australia (Western Australia (WA) and New South Wales (NSW)), Finland, Norway and the USA (California) (table 2).

**Table 2:** Description of cohort and data sources used to identify a cohort of births in four high-income countries

Location	California, USA	Finland	Norway	New South Wales, Australia	Western Australia, Australia
Time period	1991–2010	1987–2017	1980–2016	1994–2016	1980–2015
Data source	Office of Statewide Health Planning and Development (OSHPD)	National Institute for Health and Welfare (THL), Medical Birth Register	Norwegian Institute of Public Health, Medical Birth Registry	NSW Perinatal Data Collection, NSW Ministry of Health	WA Midwives Data Collection, WA Department of Health
Information available	Maternal characteristics and health conditions; smoking and BMI (2007–2010); pregnancy and labour conditions, antenatal hospitalisations, information on delivery and birth outcomes; gestational age based on LMP estimate (obstetric estimate available for years 2007–2010)	Maternal characteristics and health conditions; smoking; pregnancy and labour complications; pregnancy history; details of antenatal care; information on delivery and birth outcomes; health of infant at discharge or 7 days	Maternal characteristics and health conditions; pregnancy and labour complications; medication use during pregnancy; birth outcomes; diagnoses of congenital abnormalities; parental occupation and smoking; births following assisted conception	Maternal characteristics and health conditions; smoking; pregnancy and labour complications; details of labour; birth outcomes; congenital anomalies, infant and child health outcomes	Maternal characteristics and health conditions; smoking; pregnancy and labour complications; details of labour; birth outcomes; congenital anomalies, infant and child health outcomes
Scope of notified births	All live births and stillbirths with gestational length ≥20 weeks	All live births and stillbirths with gestational length ≥22 weeks or birth weight ≥500 g	All pregnancies ending after week 12 from 2002 onwards (from week 16 from 1980 to 2001)	Gestational length ≥20 weeks or birth weight ≥400 g	Gestational length ≥20 weeks or birth weight ≥400 g
Linkage methods	Probabilistic linkage based on maternal descriptors	Deterministic linkage of mother based on personal ID	Deterministic linkage of mother based on personal ID	Probabilistic linkage based on maternal descriptors	Probabilistic linkage based on maternal descriptors
Total births	10.9 million	1.8 million	2.1 million	2.2 million	1.2 million
Benchmark neonatal morbidity indicators	10.5% PTB (LMP estimate) 6.4% LBW	5.8% PTB 4.3% LBW	5.7% PTB 3.4% LBW	5.4% PTB 4.4% LBW	5.3% PTB 5.3% LBW

BMI, body mass index; LBW, low birth weight; LMP, last menstrual period; PTB, preterm birth.

We will conduct analyses using unmatched (all women) and matched approaches. By matching pregnancies to the same women, we will account for individual-level confounders (known and unknown) that remain stable between pregnancies. The effect of IPI from matched and unmatched analyses will be compared. The study’s population will consist of more than 18 million births, which will be the largest IPI study for high-income countries. The size of this cohort offers several advantages, as it allows us to: (1) investigate IPI effects among subpopulations, and (2) ensure the best possible control for confounding. Where within-mother matching is not possible (eg, when studying effect modification, sample size in individual data categories becomes reduced), we will match on propensity scores.^44^

### Inclusion criteria

Women with two or more consecutive pregnancies will be included in the study. The target population for inference includes women with >1 birth during their life course. IPI is not relevant for women who have only one birth. Given that matching pregnancies to the same women requires >1 pregnancy interval per woman, the cohort for matched analyses will be restricted to women who have two or more births during the study period. The 20-year to 30-year study period ensures sufficient follow-up to observe final family size. Birth status (live born vs stillborn), plurality (singleton vs multiple) and maternal age will inform inclusion criteria for specific hypotheses.

### Definition of IPI

IPI will be defined as the length of time between the start of the index pregnancy (birth date minus gestational length) and the birth date of the preceding pregnancy. IPI will be classified using integer months and categorised as: 0–5, 6–11, 12–17, 18–23, 24–59, 60–119 and ≥120 months, with 18–23 months used as the referent group.^26^ These intervals share endpoints consistent with recommendations of past studies and WHO recommendations. In addition, IPI will be considered as a continuous variable for assessment of optimal IPI.

### IPI-relevant health outcomes

Based on a review of the current literature, we identified a set of clinically significant IPI-relevant endpoints, contexts and competing risks that can be investigated accurately and reliably (see online supplementary table S1). We classified the primary IPI-relevant endpoints into the following two categories: PTB; and fetal growth restriction based on the definitions of SGA and LBW. Additional endpoints include congenital anomaly; complications of pregnancy and labour; infant and child health outcomes; and perinatal, infant and maternal mortality.

10.1136/bmjopen-2018-027941.supp1 Supplementary file 1

### Data sources

**Figure 2:**
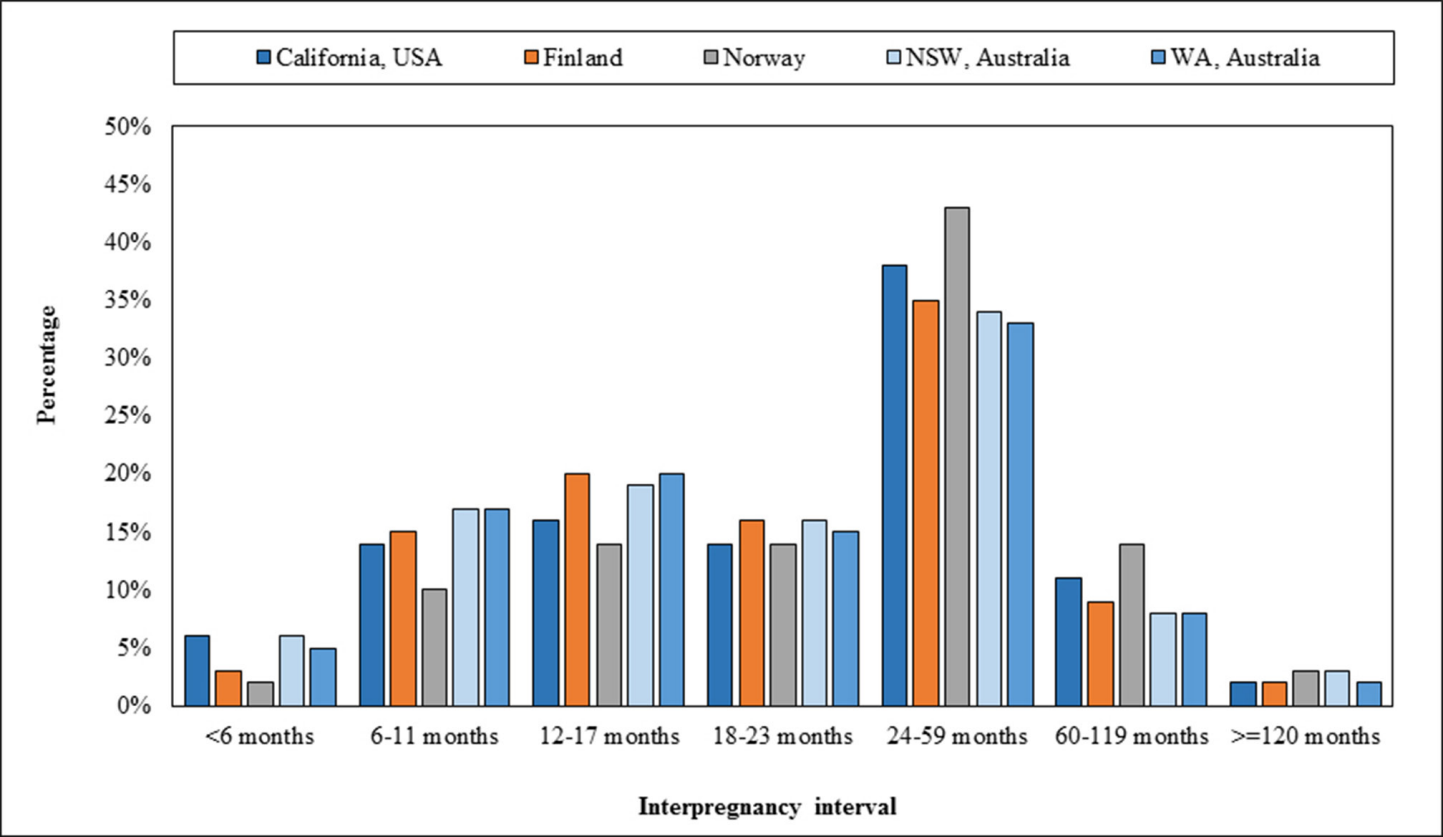
Distribution of interpregnancy interval following a live birth, by country/state—1980–2016.

Cohorts will be assembled from linked birth cohort files in California with Office of Statewide Health Planning and Development maternal and infant hospital discharge data (USA: 1991–2010); the National Institute for Health and Welfare (THL), Medical Birth Register (Finland: 1987–2017); the Norwegian Institute of Public Health, Medical Birth Registry (Norway: 1980–2015); the NSW Perinatal Data Collection, NSW Ministry of Health (Australia: 1994–2016); and the WA Midwives Data Collection, WA Department of Health (Australia: 1980–2015) (table 2). Coverage is >99% of births for each country/jurisdiction. IPIs for each data source will be derived (figure 2), and maternal characteristics and health conditions, pregnancy complications, details of labour and childbirth, birth outcomes and mortality will be identified (see online supplementary table S1). Some sites will have access to additional linked hospital and child development data, offering the opportunity to explore the impact on additional maternal and child health outcome measures, including congenital anomalies (WA, NSW, Norway, Finland) and hospital admissions (WA, NSW, Finland).

### Data deidentification and secure storage

Deidentified data from California and Finland will be retained and held securely at those sites; deidentified data from Norway, WA and NSW will be transferred to and stored at the School of Public Health, Curtin University. Project data will be electronically stored on a secure server, which is backed up daily to prevent any unintentional data loss. The research environment includes a variety of security controls to restrict unauthorised access—these include access controls, role-based delegations, encryption, firewalls and physical access restrictions (authorised access to server rooms and research offices is restricted by key). Automatic screen locking will occur on electronic devices after 5 min of inactivity. Data will not be stored or used in public terminals. No paper-based or portable electronic media storage of data will take place.

### Statistical methods

Matched and unmatched logistic regression will be used to investigate all maternal and child health outcomes (see online supplementary table S1). For primary analyses, mother ID will be used as the matching variable to identify strata. This method ensures that the results will be based entirely on within-women (not between-women) comparisons, minimising the need for additional adjustment and is the standard statistical method for matched studies. We will explicitly adjust for factors of the index pregnancy that do/can change between pregnancies. Specifically, we will adjust for maternal age (categorical variable: 14–19, 20–24, 25–29, 30–34, 35–39 and ≥40 years), parity (categorical) and conception year (non-linear spline). Due to differences between countries in the completeness and method of measurement of SES (or maternal education as a proxy), adjustment for SES will not be possible when data are pooled across countries, but may be undertaken when analyses are conducted using single-country data sets.

Matched analyses will be undertaken to address secondary aims, where possible. In circumstances where within-mother matching is not feasible or not possible (eg, subpopulations with small sample sizes which would be further restricted by requiring more than two consecutive pregnancies per mother), unmatched models or alternative matching methods will be used (eg, propensity score stratification; sibling-matched methods using a single IPI).^8 44 45^

Analyses will first be applied to each site’s data individually. The analytic approach and statistical code used for data sets held at Curtin University (WA, NSW, Norway) will be sent to the other sites (California, Finland) to replicate analyses, allowing standardisation and minimising methodological heterogeneity between countries.^46^ Meta-analysis^47^ will be used to combine adjusted ORs obtained from each data source into a single pooled estimate generalisable to high-income countries. Heterogeneity between countries will be investigated with the I^2^ statistic.^48^

### Patient and public involvement

A reference group of consumer health representatives (Healthy Pregnancies Reference Group) has been established, and will meet twice-yearly to provide a community perspective on this research. The reference group will provide advice regarding the aims of the research; language, including lay summaries; links between consumers, the community and the researchers; and advocacy on behalf of consumers and the community. The reference group will also contribute consumer perspectives on potential utilisation of the research findings, such as the identification of factors that may influence IPI (see online supplementary table S2), with the intention to inform further primary research in this area.

10.1136/bmjopen-2018-027941.supp2 Supplementary file 2

## Ethics and dissemination

### Human research ethics committee approval

This research has ethics approval from ethics committees at participating sites. Each committee provided a waiver of consent for participants. For Finland, ethical approval was not required.

### Intended publications and research dissemination

Data sets generated and/or analysed during the current study are not publicly available due to data confidentiality agreements with data custodians. Results generated by the research will be made publicly available at the summary level. Standalone manuscripts addressing primary and secondary aims will be published in peer-reviewed journals. Results will also be presented at relevant perinatal and epidemiological conferences. Findings will be used to develop guidelines for IPI as a potentially modifiable risk factor linked to adverse perinatal outcomes, specifically for women in high-income countries.

## Discussion

This study will inform new IPI recommendations for women in high-income countries. Recommendations will be specific to obstetric and socioeconomic context. By combining record-linked perinatal data from multiple countries—Australia, Finland, Norway and the USA—this study will achieve the largest sample of high-quality health data in high-income countries. Novel methodology, matching pregnancies to the same women, will achieve the best control for confounding of any study to date.

This international cohort approach offers several advantages. Large sample sizes are required to undertake matched analyses with adequate statistical power to detect clinically meaningful differences between IPIs. Prospective studies of this size are infeasible. This study can only be achieved using linked perinatal data within an international collaboration. Collaboration to standardise methods between countries will also minimise methodological heterogeneity in pooled analyses. Furthermore, this research has the potential to be extended by broadening the collaboration to incorporate additional data sets. The minimum data requirements for contributing to this project are: (1) unique identifier for mothers to link multiple pregnancies; (2) mother’s date of birth; (3) child’s date of birth; (4) child’s gestational length; and (5) other birth and early childhood outcomes as described in online supplementary table S1. Researchers interested in joining this collaboration are encouraged to contact us through the corresponding author.

This information is essential for supporting family planning, both at the patient and provider level. Evidence-based recommendations for IPI for women in high-income countries are useful for assisting families when planning future pregnancies and for clinicians when providing intrapregnancy counselling.

## Supplementary Material

Reviewer comments

Author's manuscript
