# Supplementary material for: Developing evidence-based recommendations for optimal interpregnancy intervals in high-income countries: protocol for an international cohort study
Source: BMJ Open. 2019 Jan 29;9(1):e027941. doi: 10.1136/bmjopen-2018-027941 (PMC6352763; doi:10.1136/bmjopen-2018-027941)
Supplement: Supplementary file 1 [file bmjopen-2018-027941supp001.pdf]

Table S1. Description of outcome ascertainment related to interpregnancy intervals in high-income countries and data sources for measuring outcomes.

**Category**	**Measure/Data source**	**Description**
Preterm birth	Length of gestation (completed weeks)	The quality of gestational length data has improved over time - for all cohorts, frequency of antenatal ultrasounds for pregnancy dating has increased, with corresponding declines in recording gestational lengths based on last menstrual period. PTB will be defined as all births <37 completed weeks gestation, and also sub-classified as idiopathic (spontaneous) and iatrogenic (medically indicated).
Fetal growth restriction based on SGA and low birthweight	Birth weight (grams)	LBW will be defined as weight <2500g. Birth weights are accurately measured by clinical personnel, with near-complete coverage. SGA will be defined as birth weight <10th centile (for gestational length and infant sex) of the birth weight distribution, by calendar year (which accounts for cohort effects), and by cohort (which accounts for differences between countries).
Congenital anomaly	WA, WA Register of Developmental Anomalies (WARDA)<br><br>NSW, Register of Congenital Conditions (RoCC)<br><br>Norway, Medical Birth Registry<br><br>Finland, Register of Congenital Malformations	The WARDA is a comprehensive database of all birth defects diagnosed up to 6 years of age, notifiable by legislation. A 5-year post-birth follow-up period will be used to identify birth defects. For NSW, the RoCC includes all infants diagnosed up to one year of age and only for a five-year period. The comprehensive case identification in WA will enable us to quantify non-identification of cases in the other cohorts, and weight the analyses accordingly.[50] For all cohorts combined, we estimate that >94% of cases with congenital anomaly will be identified.<br><br>In Finland, data on congenital anomalies are gathered from hospitals, health-care professionals and other national health registers. All cases recorded during pregnancy or infancy (<1 year) are included in the register. Statistical data are used for monitoring congenital anomalies nationally and regionally, and among other purposes, for planning prenatal screening, diagnostics and treatment of congenital anomalies as well as for conducting research on congenital anomalies. https://thl.fi/en/web/thlfi-en/statistics/information-on-statistics/register-descriptions/register-of-congenital-malformations.
Complications	Placental abruption, pPROM,	These databases have coverage of every birth within the state/country, and

of pregnancy and labour	labour dystocia, uterine rupture, pre-eclampsia, gestational hypertension, gestational diabetes<br><br>Midwives Data Collection (WA), Perinatal Data Collection (NSW), maternal antenatal hospitalisations (WA, NSW), birth certificates (California) and the Medical Birth Registers (Finland, Norway).	the complications of labour and pregnancy are recorded at birth. For California, ICD coded complications (ICD-9) of pregnancy and labour that resulted in antenatal hospitalisation are additionally included on the birth certificate records themselves, and will be used to supplement case identification. For WA, NSW and Finland, case identification will be supplemented with a search of the following primary and secondary diagnosis codes (ICD-9/10) of all hospital admissions between start of pregnancy and the discharge date of the birth admission of the mother. Placental abruption: 641.2, O45; labour dystocia: 662.0, O63.0; uterine rupture: 665.1, O71.0, O71.1; pre-eclampsia: 642.4, 642.5, 642.7, O14; gestational hypertension: 642.3, O13; pPROM: 658.1, 658.2 (+ gestational length <37wks), O42; gestational diabetes: 648.8, O24.4
Infant and child health outcomes	Hospital admissions (WA, NSW, Finland)	Hospital contacts will identified from hospital admissions (WA, NSW, Finland) with 3-year follow-up (birth-age 3 years).
Perinatal, infant and maternal mortality	Death registrations (WA, NSW, Norway, Finland) and death certificates (California).	These data sources record every death registered in the state/country. The date of death will be utilised to identify stillbirths (week 20 gestation – birth), perinatal mortality (stillbirths + neonatal mortality), infant mortality (28 days – age 1 year) early childhood mortality (age 1 year to age 3 years), and maternal mortality (pregnancy period – 42 days postpartum). Accidental death, homicide, and other external causes will be identified with cause of death codes and subsequently excluded. Hospital contacts will identified from hospital admissions (WA, NSW, Finland) with 3-year follow-up (birth-age 3 years) and investigated as binary outcomes (>0; >10th,25th,50^{th}centiles of frequency).
