# Supplementary material for: Developing evidence-based recommendations for optimal interpregnancy intervals in high-income countries: protocol for an international cohort study
Source: BMJ Open. 2019 Jan 29;9(1):e027941. doi: 10.1136/bmjopen-2018-027941 (PMC6352763; doi:10.1136/bmjopen-2018-027941)
Supplement: Supplementary file 2 [file bmjopen-2018-027941supp002.pdf]

Table S2: Factors affecting pregnancy intervals identified by our Healthy Pregnancy Consumer Reference Group.

Psychological factors	*Replace* a pregnancy loss; compensate for a previous adverse birth outcome; psychological preparation – fear of pregnancy loss or adverse birth outcome in the previous pregnancy; sexual activity is a reminder of pregnancy loss, disability or other adverse birth outcome experienced; relationship stability – partner unsupportive of increasing family size, relationship difficulties, no partner; postnatal depression after the first birth; depression, anxiety, stress, flare of an existing mental health problem or difficulty managing medication while pregnant; experience of a stress-event (death of a loved one, moving home, job loss, divorce/separation); reproductive coercion/abuse.
**Healthcare provision**	Lack of information from healthcare professionals as to clinical recommendations on birth spacing, and their associated risks; encouragement by obstetricians to try again for a child soon after pregnancy loss; information GPs that breastfeeding prevents pregnancy without provision of guidance; no information provided by specialists on risks associated with timing of pregnancy.
**Lifestyle factors/choices**	*Not miss out* - experience what they had planned but could not experience in the first pregnancy; advanced parental age; sex balance desired in family – trying for a girl/boy; household preparation – residential mobility, physical space in the home; ability to manage the day-to-day care of the current child or children – with/without first child morbidities; maternal career aspirations; physical preparation to improve fitness and health; desire to replicate/avoid age gaps in family members; change in partner; multiple gestation in first pregnancy.
**Support and social network**	Availability of support from family – childcare; living in a region distant from extended family; couple living/working in different regions from each other; increase in family size of friends and family; interference from parents/in-laws in the marriage or parenting.
**Contraception sexual activity Fecundity**	Lack of contraceptive use or proper contraceptive use; lack of knowledge regarding pregnancy planning e.g., breast feeding required lactational amenorrhea; failed contraception; higher levels of sexual activity; couple living/working away or work schedules clashing (e.g., shift work); difficulty falling pregnant; reproductive treatment; failed treatment; delays in treatment.
**Financial factors**	Higher paternal income; lower paternal income or reliance on maternal income; cost of contraception; financial preparation – savings, debt; cost of fertility treatment; cost of childcare – more than one child in childcare at the same time is financially prohibitive; paid maternity leave requirements e.g., returning to work for at least 1 year to become eligible.
**Morbidity Healing**	Major morbidities including heart disease, malignant neoplasm; known recurrent pregnancy issues (e.g., hyperemesis gravidarum); healing time required after illness or the previous pregnancy; fear of effects of medication on the fetus; hormonal therapy (progesterone, HCG).
