# Supplementary material for: Developing evidence-based recommendations for optimal interpregnancy intervals in high-income countries: protocol for an international cohort study
Source: BMJ Open. 2019 Jan 29;9(1):e027941. doi: 10.1136/bmjopen-2018-027941 (PMC6352763; doi:10.1136/bmjopen-2018-027941)
Supplement: Author's manuscript [file bmjopen-2018-027941.draft_revisions.pdf]

BMJ Open is committed to open peer review. As part of this commitment we make the peer review history of every article we publish publicly available.

When an article is published we post the peer reviewers' comments and the authors' responses online. We also post the versions of the paper that were used during peer review. These are the versions that the peer review comments apply to.

The versions of the paper that follow are the versions that were submitted during the peer review process. They are not the versions of record or the final published versions. They should not be cited or distributed as the published version of this manuscript.

BMJ Open is an open access journal and the full, final, typeset and author-corrected version of record of the manuscript is available on our site with no access controls, subscription charges or pay-per-view fees (http://bmjopen.bmj.com).

If you have any questions on BMJ Open's open peer review process please email editorial.bmjopen@bmj.com

BMJ Open

Developing evidence-based recommendations for optimal interpregnancy intervals in high-income countries: protocol for an international cohort study

Journal:	*BMJ Open*
Manuscript ID	bmjopen-2018-027941
Article Type:	Protocol
Date Submitted by the Author:	16-Nov-2018
Complete List of Authors:	Marinovich, M Luke; Curtin University, School of Public Health<br>Regan, Annette; Texas A&M University, School of Public Health<br>Gissler, Mika; THL National Institute for Health and Welfare, Information<br>Magnus, Maria; University of Bristol, MRC Integrative Epidemiology Unit; Norwegian Institute of Public Health, Centre for Fertility and Health (CeFH)<br>Håberg, Siri; Norwegian Institute of Public Health,<br>Padula, Amy; University of California San Francisco Department of Obstetrics Gynecology and Reproductive Sciences,<br>Mayo, Jonathan; Stanford University, Department of Pediatrics<br>Shaw, Gary M.; Stanford University, Department of Pediatrics<br>Ball, Stephen; Curtin University, Prehospital, Resuscitation and Emergency Care Research Unit (PRECRU), School of Nursing, Midwifery and Paramedicine<br>malacova, eva; Curtin University, School of Public Health<br>Gebremedhin, Amanuel; Curtin University, School of Public Health<br>Nassar, Natasha; University of Sydney, Menzies Centre for Health Policy, School of Public Health<br>Marston, Cicely; London School of Hygiene and Tropical Medicine, Faculty of Public Health and Policy<br>de Klerk, Nick; University of Western Australia, Telethon Kids Institute<br>Betran, Ana Pilar; World Health Organization, Department of Reproductive Health and Research<br>Pereira, G.F; Curtin University, School of Public Health
Keywords:	pregnancy, birth intervals, family planning, siblings, preterm birth, fetal growth restriction

SCHOLARONE™ Manuscripts

Developing evidence-based recommendations for optimal interpregnancy intervals in high-income countries: protocol for an international cohort study

M Luke Marinovich,[1] Annette K Regan,[2] Mika Gissler,[3,4] Maria C Magnus,[5,6,7] Siri Håberg,[7] Amy M Padula,[8] Jonathan A Mayo,[9] Gary M Shaw,[9] Stephen Ball,[10] Eva Malacova,[1] Amanuel T Gebremedhin,[1] Natasha Nassar,[11] Cicely Marston,[12] Nick de Klerk,[13] Ana Pilar Betrán,[14] Gavin Pereira[1]

Author affiliations:

[1] School of Public Health, Curtin University, Perth, Western Australia, Australia

[2] School of Public Health,Texas A&M University, College Station, Texas, USA

[3] Information Services Department, National Institute for Health and Welfare, Helsinki, Finland

[4] Department of Neurobiology, Care Sciences and Society, Karolinska Institute, Stockholm, Sweden

[5] MRC Integrative Epidemiology Unit at the University of Bristol, Bristol, United Kingdom

[6] Population Health Sciences, Bristol Medical School, Bristol, United Kingdom

[7] Centre for Fertility and Health (CeFH), Norwegian Institute of Public Health, Oslo, Norway

[8] Department of Obstetrics, Gynecology and Reproductive Sciences, University of California, San Francisco, USA

[9] Department of Pediatrics, Stanford University, Stanford, USA

[10] School of Nursing, Midwifery and Paramedicine, Curtin University, Perth, Western Australia, Australia

[11] Menzies Centre for Health Policy, School of Public Health, University of Sydney, Sydney, New South Wales, Australia

[12] Faculty of Public Health and Policy, London School of Hygiene and Tropical Medicine, London, UK

[13] Telethon Kids Institute, University of Western Australia, Subiaco, Western Australia, Australia

[14] UNDP/UNFPA/UNICEF/WHO/World Bank Special Programme of Research, Development and Research Training in Human Reproduction, Department of Reproductive Health and Research, World Health Organization, Geneva, Switzerland

Corresponding author:

Dr ML Marinovich, Research Fellow

School of Public Health, Curtin University

GPO Box U1987

Perth Western Australia 6845

Email: Luke.Marinovich@curtin.edu.au

Phone: +61 8 9266 4006

Protocol version: 1.0 (November 2018)

Word count: 2735

For peer review only - http://bmjopen.bmj.com/site/about/guidelines.xhtml

ABSTRACT

Introduction: Short interpregnancy interval (IPI) has been linked to adverse pregnancy outcomes. The World Health Organization (WHO) recommends waiting at least two years after a live birth and six months after miscarriage or induced termination before conception of another pregnancy. The evidence underpinning these recommendations largely relies on data from low and middle-income countries. Furthermore, recent epidemiological investigations have suggested that these studies may over-estimate the effects of IPI due to residual confounding. Future investigations of IPI effects in high-income countries drawing from large, population-based data sources are needed to inform IPI recommendations. We aim to assess the impact of IPIs on maternal and child health outcomes in high-income countries.

Methods and Analysis: This international longitudinal retrospective cohort study will include more than 18 million pregnancies, making it the largest study to investigate IPI in high-income countries. Population-based data from Australia, Finland, Norway, and US will be utilised. Birth records in each country will be used to identify consecutive pregnancies. Exact dates of birth and clinical best estimates of gestational length will be used to estimate IPI. Administrative birth and health data sources with >99% coverage in each country will be used to identify maternal sociodemographics, pregnancy complications, details of labour and delivery, birth and child health information. We will use matched and unmatched regression models to investigate the impact of IPI on maternal and infant outcomes, and conduct meta-analysis to pool results across countries.

Ethics and Dissemination: Ethics boards in at participating sites approved this research (approval was not required in Finland). Findings will be published in peer-reviewed journals and presented at international conferences, and will inform recommendations for optimal IPI in high-income countries. Findings will provide important information for women and

For peer review only - http://bmjopen.bmj.com/site/about/guidelines.xhtml

families planning future pregnancies and for clinicians providing prenatal care and giving guidance on family planning.

Keywords: pregnancy; birth intervals; family planning; siblings; preterm birth; fetal growth restriction

For peer review only

ARTICLE SUMMARY

Strengths and limitations of this study

- With data from four countries and over 18 million pregnancies, this will be the largest cohort study to investigate IPI in high-income countries.
- The size of this cohort will allow strict control for confounding through matching multiple pregnancies within women, and permit the investigation of IPI effects among sub-populations.
- Collaboration between countries and the use of individual-level data will minimise methodological heterogeneity.
- Data items such as socioeconomic status vary between countries in their completeness and their method of measurement, limiting the extent to which such variables may be incorporated as covariates.
- Data on early pregnancy loss are poorly or inconsistently captured in routine data collection, which may result in misclassification of IPI.

For peer review only

INTRODUCTION

Interpregnancy interval (IPI), or the time from birth to conception of the next pregnancy, has been identified as a potentially modifiable risk factor linked to adverse perinatal outcomes. Currently, the World Health Organization (WHO) recommends that women wait at least two years after a live birth and six months after early pregnancy loss before conceiving again to reduce the risk of adverse maternal and perinatal outcomes.[1] This recommendation is based on observational studies, mostly from low and middle-income countries, that have demonstrated associations between short IPI and adverse pregnancy outcomes,[2] most notably maternal mortality,[3] small for gestational age (SGA), term low birth weight (LBW),[4] preterm pre-labour rupture of membranes (pPROM),[5,6] preterm birth (PTB)[4,7,8] and birth defects.[9,10] Furthermore, long IPI has been shown to be associated with higher risk of outcomes such as fetal death,[11] LBW, PTB, SGA,[12] and pre-eclampsia[11,13].

Several theories have been proposed to explain these associations. Short IPIs may leave insufficient time to recover from maternal nutrient deficits, which can lead to fetal-maternal competition for essential nutrients.[14] This can be exacerbated by lactation[15] and malnourishment, in both high and low-income countries.[16,17] Short IPIs might also leave insufficient recovery time from inflammatory processes from the previous pregnancy that extend into the next pregnancy.[2] Long IPIs may result in the loss of adaptive benefits to the mother from a previous birth, resulting in a return to a state equivalent to primigravida.[17] An alternative hypothesis is that the observed associations between IPI and adverse pregnancy outcomes can be explained, at least partially, by systematic bias.[18] A spurious association would result if time to conception were independently associated with other factors causally linked to adverse pregnancy outcomes in the subsequent pregnancy.[19,20] For example, patterns in IPI are associated with maternal age, socioeconomic status (SES),

For peer review only - http://bmjopen.bmj.com/site/about/guidelines.xhtml

breastfeeding, and other antenatal, postnatal, or postpartum practices.[21,22] Furthermore, many factors that can confound the associations between IPI and adverse pregnancy outcomes, such as psychosocial determinants of health, are difficult to measure. Undoubtedly, the greatest challenge for observational studies is to comprehensively account for such confounding factors. High quality studies have adjusted for potential confounders, but statistical adjustment is rarely complete. Residual unmeasured confounding can lead to bias[23] of the observed effect of IPI on adverse pregnancy outcomes. Importantly, as the mechanisms by which IPI affects specific pregnancy outcomes are not well-understood, direct adjustment cannot be made for confounders that remain unknown. Under this scenario, the current state of knowledge on the effects of IPI is clinically unreliable.

In recent years, studies have attempted to address the issue of bias from unmeasured confounding through a matched longitudinal study design.[24-26] Longitudinal study designs which match pregnancies to the same women[26,27] can better overcome confounding introduced by unknown, unmeasured and inaccurately measured factors that tend to vary between mothers but remain similar between pregnancies.[24,25,28] Findings from these studies suggest the effects of IPI reported from unmatched models are over-estimated. Considerable reductions in the U-shape effect of IPI are observed after matching pregnancies to the same women (Figure 1),[26] indicating support for the systematic bias hypothesis. These results provide a persuasive argument that some of the IPI effect observed in past studies may be attributable to confounders that vary between women (e.g., heritable/genetic, socioeconomic, recurrent health-related behaviour, unknown/unmeasured), but can be effectively controlled under a longitudinal matched design.

Although these results cast doubt over the adverse effects of IPI, the existence of adverse associations with perinatal outcomes and the optimal IPI period remains unclear due to smaller sample sizes for matched analyses. Confidence intervals around estimates are

For peer review only - http://bmjopen.bmj.com/site/about/guidelines.xhtml

typically wider for matched analyses which rely on women having at least two IPIs during the study period, particularly for the effects of longer IPI (>60 months). In addition, the selection of women with more than two pregnancies in matched analyses limits generalisability to all women. Several other gaps remain in our knowledge around IPI effects (Table 1). First, the majority of studies have so far been restricted to low and middle-income countries. For example, the current WHO recommendation for IPI following early pregnancy loss are based on the findings from a single, albeit large, study in Latin America,[4] and it remains unclear as to whether the findings are relevant to high-income countries. Second, some of the most pertinent questions for women in high-income countries, such as those related to obstetric context, have not yet been addressed. Additional data from high-income countries and more robust results are needed.

Study aims

The primary aim of this study is to evaluate the impact of IPI on pregnancy outcomes in high-income countries. Secondary aims are to:

1. **Evaluate the impact of IPI based on obstetric context:** IPI consequences may differ for women based on obstetric history. For example, optimal IPI may vary for women who previously gave birth by caesarean section due to risk of uterine rupture after insufficient time for the uterine scar to heal.[29] The effect of IPI may also vary depending on gestational age of the previous birth, since longer gestation could be expected to prolong the maternal nutrient deficits and inflammatory processes implicated in poorer outcomes for the subsequent birth, particularly with short IPI. Furthermore, recommendations of IPI after stillbirth require specific attention[30]. Pregnancy intervals for women following a stillbirth or neonatal loss are approximately one year shorter than intervals for women without a loss.[31,32] It has been speculated that this is due to parents wishing to counter the emotional loss of

For peer review only - http://bmjopen.bmj.com/site/about/guidelines.xhtml

their infant or to minimise the delay in having a baby.[33] However, a survey of obstetricians regarding the timing of pregnancy following pregnancy loss found approximately two thirds of obstetricians endorse women waiting less than 6 months after stillbirth before trying to conceive a new pregnancy.[34] This is incongruous with the WHO IPI recommendations that women wait at least 6 months after pregnancy loss.

2. **Evaluate the impact of IPI based on socio-demographic context:** The IPI recommendations generated by this study may be most beneficial for those populations for whom we know short IPI and high parity (large family size) is prevalent. Within high-income countries, both family size and the IPI distribution can vary markedly according to race/ethnicity and SES.[35,36] In this study, we will investigate the effects of IPI among women from different socioeconomic and racial/ethnic groups in multiple high-income countries.

3. **Evaluate the potential influence of maternal age on the effect of IPI on pregnancy outcomes:** In high income countries, age at childbirth overall is increasing, with later age at first birth.[37-39] Systematic reviews conclude that increased maternal age is associated with greater risk of a wide range of adverse obstetric outcomes,[40] including perinatal mortality,[41] PTB and LBW,[42] and pregnancy intervention, particularly caesarean section.[43] In this study, we will test the hypothesis that there is an interaction between increased maternal age and IPI on the risk of adverse pregnancy outcomes. For each year of maternal age (i.e. maternal age at first observed birth in the study period) and for different IPIs, we will calculate the risk of adverse pregnancy outcomes. This will provide valuable information on

For peer review only - http://bmjopen.bmj.com/site/about/guidelines.xhtml

any combined risks of maternal age and IPI for older women conceiving their second (or later) child.

METHODS AND ANALYSIS

Study design and population

We will conduct a longitudinal retrospective cohort study on the effects of IPI on maternal and child health outcomes using individual-level records from with near complete coverage of all births (>99%) in Australia (Western Australia [WA] and New South Wales [NSW]), Finland, Norway, and the United States (US; California) (Table 2).

We will conduct analyses using unmatched (all women) and matched approaches. By matching pregnancies to the same women, we will account for individual-level confounders (known and unknown) that remain stable between pregnancies. The effect of IPI from matched and unmatched analyses will be compared. The study's population will consist of more than 18 million births, which will be the largest IPI study for high-income countries. The size of this cohort offers several advantages, as it allows us to: a) investigate IPI effects among sub-populations, and b) ensure the best possible control for confounding. Where within-mother matching is not possible (e.g. when studying effect-modification, sample size in individual data categories becomes reduced), we will match on propensity scores.[44]

Inclusion criteria

Women with two or more consecutive pregnancies will be included in the study. The target population for inference includes women with >1 birth during their life course. IPI is not relevant for women who have only one birth. Given that matching pregnancies to the same women requires >1 pregnancy interval per woman, the cohort for matched analyses will be restricted to women who have 2 or more births during the study period. The 20 to 30-year

For peer review only - http://bmjopen.bmj.com/site/about/guidelines.xhtml
For peer review only

study period ensures sufficient follow-up to observe final family size. Birth status (live born vs stillborn), plurality (singleton vs multiple) and maternal age will inform inclusion criteria for specific hypotheses.

Definition of IPI

IPI will be defined as the length of time between the start of the index pregnancy (birth date minus gestational length) and the birth date of the preceding pregnancy. IPI will be classified using integer months and categorised as: 0-5, 6-11, 12-17, 18-23, 24-59, 60-119, and ≥120 months, with 18-23 months used as the referent group.[26] These intervals share endpoints consistent with recommendations of past studies and WHO recommendations. In addition, IPI will be considered as a continuous variable for assessment of optimal IPI.

IPI-relevant health outcomes

Based on a review of the current literature, we identified a set of clinically significant IPI-relevant endpoints, contexts and competing risks that can be investigated accurately and reliably (Table S1). We classified the *primary* IPI-relevant endpoints into the following two categories: preterm birth (PTB); and fetal growth restriction based on the definitions of small for gestational age (SGA) and low birthweight (LBW). Additional endpoints include congenital anomaly; complications of pregnancy and labour; infant and child health outcomes; and perinatal, infant and maternal mortality.

Data sources

Cohorts will be assembled from linked birth cohort files in California with Office of Statewide Health Planning and Development maternal and infant hospital discharge data (USA: 1991-2010); the National Institute for Health and Welfare (THL), Medical Birth Register (Finland: 1987-2017); the Norwegian Institute of Public Health, Medical Birth Registry (Norway: 1980-2015); the NSW Perinatal Data Collection, NSW Ministry of Health

For peer review only - http://bmjopen.bmj.com/site/about/guidelines.xhtml

(Australia: 1994-2016); and the WA Midwives Data Collection, WA Department of Health (Australia: 1980-2015) (Table 2). Coverage is >99% of births for each country/jurisdiction. IPIs for each data source will be derived (Figure 2), and maternal characteristics and health conditions, pregnancy complications, details of labour and childbirth, birth outcomes and mortality will be identified (Table S1). Some sites will have access to additional linked hospital and child development data, offering the opportunity to explore the impact on additional maternal and child health outcome measures, including congenital anomalies (WA, NSW, Norway, Finland) and hospital admissions (WA, NSW, Finland).

Data de-identification and secure storage

De-identified data from California and Finland will be retained and held securely at those sites; de-identified data from Norway, WA and NSW will be transferred to and stored at the School of Public Health, Curtin University. Project data will be electronically stored on a secure server, which is backed up daily to prevent any unintentional data loss. The research environment includes a variety of security controls to restrict unauthorised access – these include access controls, role-based delegations, encryption, firewalls, and physical access restrictions (authorised access to server rooms and research offices is restricted by key). Automatic screen locking will occur on electronic devices after five minutes of inactivity. Data will not be stored or used in public terminals. No paper-based or portable electronic media storage of data will take place.

Statistical methods

Matched and unmatched logistic regression will be used to investigate all maternal and child health outcomes (Table S1). For primary analyses, mother ID will be used as the matching variable to identify strata. This method ensures that the results will be based entirely on within-women (not between-women) comparisons, minimising the need for additional

For peer review only

adjustment and is the standard statistical method for matched studies. We will explicitly adjust for factors of the index pregnancy that do/can change between pregnancies. Specifically, we will adjust for maternal age (categorical variable: 14-19, 20-24, 25-29, 30-34, 35-39, and ≥40 years), parity (categorical), and conception year (non-linear spline). Due to differences between countries in the completeness and method of measurement of SES (or maternal education as a proxy), adjustment for SES will not be possible when data are pooled across countries, but may be undertaken when analyses are conducted using single-country datasets.

Matched analyses will be undertaken to address secondary aims, where possible. In circumstances where within-mother matching is not feasible or not possible (e.g. sub-populations with small sample sizes which would be further restricted by requiring more than 2 consecutive pregnancies per mother), unmatched models or alternative matching methods will be used (e.g. propensity score stratification; sibling-matched methods using a single IPI).[8,44,45]

Analyses will first be applied to each site's data individually. The analytic approach and statistical code used for data sets held at Curtin University (WA, NSW, Norway) will be sent to the other sites (California, Finland) to replicate analyses, allowing standardisation and minimising methodological heterogeneity between countries[46]. Meta-analysis[47] will be used to combine adjusted odds ratios (ORs) obtained from each data source into a single pooled estimate generalisable to high-income countries. Heterogeneity between countries will be investigated with the I^2 statistic.[48]

Patient and public involvement

A reference group of consumer health representatives (*Healthy Pregnancies Reference Group)* has been established, and will meet twice-yearly to provide a community perspective

For peer review only - http://bmjopen.bmj.com/site/about/guidelines.xhtml

on this research. The reference group will provide advice regarding the aims of the research; language, including lay summaries; links between consumers, the community, and the researchers; and advocacy on behalf of consumers and the community. The reference group will also contribute consumer perspectives on potential utilisation of the research findings, such as the identification of factors that may influence IPI (Table S2), with the intention to inform further primary research in this area.

ETHICS AND DISSEMINATION

Human research ethics committee approval

This research has ethics approval from the Department of Health Western Australia Human Research Ethics Committee; Curtin University Human Research Ethics Committee; NSW Population and Health Services Research Ethics Committee; Stanford University Institutional Review Board; and the Norwegian Regional Committees for Medical and Health Research Ethics. Each committee provided a waiver of consent for participants. For Finland, ethical approval was not required.

Intended publications and research dissemination

Datasets generated and/or analysed during the current study are not publicly available due to data confidentiality agreements with data custodians. Results generated by the research will be made publicly available at the summary level. Standalone manuscripts addressing primary and secondary aims will be published in peer-reviewed journals. Results will also be presented at relevant perinatal and epidemiological conferences. Findings will be used to develop guidelines for IPI as a potentially modifiable risk factor linked to adverse perinatal outcomes, specifically for women in high-income countries.

For peer review only - http://bmjopen.bmj.com/site/about/guidelines.xhtml

DISCUSSION

This study will inform new IPI recommendations for women in high-income countries. Recommendations will be specific to obstetric and socioeconomic context. By combining record-linked perinatal data from multiple countries – Australia, Finland, Norway, and the US – this study will achieve the largest sample of high-quality health data in high-income countries. Novel methodology, matching pregnancies to the same women, will achieve the best control for confounding of any study to date.

This international cohort approach offers several advantages. Large sample sizes are required to undertake matched analyses with adequate statistical power to detect clinically meaningful differences between IPIs. Prospective studies of this size are infeasible. This study can only be achieved using linked perinatal data within an international collaboration. Collaboration to standardise methods between countries will also minimise methodological heterogeneity in pooled analyses. Furthermore, this research has the potential to be extended by broadening the collaboration to incorporate additional data sets. The minimum data requirements for contributing to this project are: 1) unique identifier for mothers to link multiple pregnancies; 2) mother's date of birth; 3) child's date of birth; 4) child's gestational length; and 5) other birth and early childhood outcomes as described in Table S1. Researchers interested in joining this collaboration are encouraged to contact us through the corresponding author.

This information is essential for supporting family planning, both at the patient and provider level. Evidence-based recommendations for IPI for women in high-income countries are useful for assisting families when planning future pregnancies and for clinicians when providing intra-pregnancy counselling.

For peer review only - http://bmjopen.bmj.com/site/about/guidelines.xhtml

List of abbreviations

IPI, interpregnancy interval

LBW, low birthweight

NSW, New South Wales

OR, odds ratio

pPROM, preterm premature rupture of membranes

PTB, preterm birth

SES, socioeconomic status

SGA, small-for-gestational age

US, United States

WA, Western Australia

WHO, World Health Organization

DECLARATIONS

Ethics

This study was approved by the Department of Health Western Australia Human Research Ethics Committee; Curtin University Human Research Ethics Committee; NSW Population and Health Services Research Ethics Committee; Stanford University Institutional Review Board; and the Norwegian Regional Committees for Medical and Health Research Ethics. Each committee provided a waiver of consent for participants. For Finland, ethical approval was not required.

Consent for publication

The authors confirm this work is original and has not been published elsewhere, in part or in whole.

Competing interests

The authors have no competing interests to declare.

Funding

For peer review only

This work was supported by funding from the National Health and Medical Research Council, including an Early Career Fellowship [#GNT1138425 to AR], Senior Research Fellowship [# 1117105 to HL], Career Development Fellowship [#APP1067066 to NN] and project grants [#1099655 to GP, NN, SJB; #APP1141510 to AR, GP]. MCM works at the MRC Integrative Epidemiology Unit at the University of Bristol which receives infrastructure funding from UK Medical Research Council (MC/UU/12013/5), and she is also supported by the following fellowship from the UK Medical Research Council (MR/M009351/1). This work was partly supported by the Research Council of Norway through its Centres of Excellence funding scheme, project number 262700. The authors have no potential conflicts of interests to disclose.

Author contributions

GP, SB, AR and MLM conceived the idea, planned and designed the study protocol. MLM and AR wrote the first draft. All authors contributed to the development of the protocol, study design and methods. All authors have approved the final written manuscript.

Acknowledgements

The authors would like to thank the Linkage and Client Services Teams at the Data Linkage Branch (Department of Health Western Australia) as well as the Data Custodian for the Midwives Notification System. The authors would also like to thank members of the Healthy Pregnancies Consumer Reference Group who provided community input and guidance in this research.

For peer review only - http://bmjopen.bmj.com/site/about/guidelines.xhtml

References

1. Marston C. Report of a WHO Technical Consultation on Birth Spacing Geneva Switzerland 13-15 June 2005: World Health Organization, 2007.
2. Shachar BZ, Lyell DJ. Interpregnancy interval and obstetrical complications. Obstetrical & gynecological survey 2012;**67**(9):584-96.
3. Conde-Agudelo A, Belizán JM. Maternal morbidity and mortality associated with interpregnancy interval: cross sectional study. Bmj 2000;**321**(7271):1255-59.
4. Conde-Agudelo A, Belizán JM, Norton MH, et al. Effect of the Interpregnancy Interval on Perinatal Outcomes in Latin America. Obstetrics & Gynecology 2005;**106**(2):359-66 10.1097/01.AOG.0000171118.79529.a3.
5. Getahun D, Strickland D, Ananth CV, et al. Recurrence of preterm premature rupture of membranes in relation to interval between pregnancies. Am J Obstet Gynecol 2010;**202**(6):570. e1-70. e6.
6. Razzaque A, Da Vanzo J, Rahman M, et al. Pregnancy spacing and maternal morbidity in Matlab, Bangladesh. International Journal of Gynecology & Obstetrics 2005;**89**:S41-S49.
7. Smith GCS, Pell JP, Dobbie R. *Interpregnancy interval and risk of preterm birth and neonatal death: retrospective cohort study*, 2003.
8. Regan AK, Ball SJ, Warren JL, et al. A Population-based Matched Sibling Analysis Estimating the Association between First Interpregnancy Interval and Birth Outcomes. American journal of epidemiology 2018.
9. Grisaru-Granovsky S, Gordon E-S, Haklai Z, et al. Effect of interpregnancy interval on adverse perinatal outcomes—a national study. Contraception 2009;**80**(6):512-18.
10. Kwon S, Lazo-Escalante M, Villaran M, et al. Relationship between interpregnancy interval and birth defects in Washington State. Journal of Perinatology 2011;**32**(1):45-50.
11. Mignini LE, Carroli G, Betran AP, et al. Interpregnancy interval and perinatal outcomes across Latin America from 1990 to 2009: a large multi-country study. BJOG : an international journal of obstetrics and gynaecology 2016;**123**(5):730-37.
12. Conde-Agudelo A, Rosas-Bermúdez A, Kafury-Goeta AC. Birth spacing and risk of adverse perinatal outcomes: a meta-analysis. Jama 2006;**295**(15):1809-23.
13. Cormick G, Betrán AP, Ciapponi A, et al. Inter-pregnancy interval and risk of recurrent pre-eclampsia: systematic review and meta-analysis. Reproductive Health 2016;**13**:83.
14. Winkvist A, Rasmussen KM, Habicht J-P. A new definition of maternal depletion syndrome. American journal of public health 1992;**82**(5):691-94.
15. Adair LS, Popkin BM. Prolonged lactation contributes to depletion of maternal energy reserves in Filipino women. The Journal of nutrition 1992;**122**(8):1643-55.
16. Adam I, Ismail MH, Nasr AM, et al. Low birth weight, preterm birth and short interpregnancy interval in Sudan. Journal of Maternal-Fetal and Neonatal Medicine 2009;**22**(11):1068-71.
17. Zhu B-P, Rolfs RT, Nangle BE, et al. Effect of the interval between pregnancies on perinatal outcomes. New England journal of medicine 1999;**340**(8):589-94.
18. Miller JE. Birth intervals and perinatal health: an investigation of three hypotheses. Family planning perspectives 1991:62-70.
19. Sholapurkar S. Is there an ideal interpregnancy interval after a live birth, miscarriage or other adverse pregnancy outcomes? Journal of Obstetrics & Gynaecology 2010;**30**(2):107-10.
20. Thom DH, Nelson LM, Vaughan TL. Spontaneous abortion and subsequent adverse birth outcomes. Am J Obstet Gynecol 1992;**166**(1):111-16.
21. Flacking R, Nyqvist KH, Ewald U. Effects of socioeconomic status on breastfeeding duration in mothers of preterm and term infants. The European Journal of Public Health 2007;**17**(6):579-84.
22. Blakely T, Hunt D, Woodward A. Confounding by socioeconomic position remains after adjusting for neighbourhood deprivation: an example using smoking and mortality. Journal of Epidemiology and Community Health 2004;**58**(12):1030-31.

For peer review only - http://bmjopen.bmj.com/site/about/guidelines.xhtml

23. Kaufman JS, Cooper RS, McGee DL. Socioeconomic Status and Health in Blacks and Whites: The Problem of Residual Confounding and the Resiliency of Race. Epidemiology 1997;**8**(6):621-28.
24. Shachar BZ, Mayo JA, Lyell DJ, et al. Interpregnancy interval after live birth or pregnancy termination and estimated risk of preterm birth: a retrospective cohort study. BJOG : an international journal of obstetrics and gynaecology 2016;**123**(12):2009-17.
25. Hanley GE, Hutcheon JA, Kinniburgh BA, et al. Interpregnancy Interval and Adverse Pregnancy Outcomes: An Analysis of Successive Pregnancies. Obstetrics and gynecology 2017;**129**(3):408-15.
26. Ball SJ, Pereira G, Jacoby P, et al. Re-evaluation of link between interpregnancy interval and adverse birth outcomes: retrospective cohort study matching two intervals per mother. BMJ 2014;**349**:g4333.
27. Pereira G, Bell ML, Lee HJ, et al. Sources of Fine Particulate Matter and Risk of Preterm Birth in Connecticut, 2000–2006: A Longitudinal Study. Environ Health Perspect 2014.
28. Susser E, Eide MG, Begg M. Invited Commentary: The Use of Sibship Studies to Detect Familial Confounding. American journal of epidemiology 2010;**172**(5):537-39.
29. Conde-Agudelo A, Rosas-Bermúdez A, Kafury-Goeta AC. Effects of birth spacing on maternal health: a systematic review. Am J Obstet Gynecol 2007;**196**(4):297-308.
30. Regan AK, Gissler M, Magnus MC, et al. Adverse birth outcomes associated with interpregnancy interval among women with a previous stillbirth: an international cohort study. Lancet in press.
31. August EM, Salihu HM, Weldeselasse H, et al. Infant mortality and subsequent risk of stillbirth: a retrospective cohort study. BJOG : an international journal of obstetrics and gynaecology 2011;**118**(13):1636-45.
32. Black M, Shetty A, Bhattacharya S. Obstetric outcomes subsequent to intrauterine death in the first pregnancy. BJOG : an international journal of obstetrics and gynaecology 2008;**115**(2):269-74.
33. Rowland Hogue CJ, Silver RM. Racial and ethnic disparities in United States: stillbirth rates: trends, risk factors, and research needs. Seminars in perinatology 2011;**35**(4):221-33.
34. Gold KJ, Leon I, Chames MC. National survey of obstetrician attitudes about timing the subsequent pregnancy after perinatal death. Am J Obstet Gynecol 2010;**202**(4):357.e1-57.e6.
35. Hogue CJ, Menon R, Dunlop AL, et al. Racial disparities in preterm birth rates and short inter-pregnancy interval: an overview. Acta obstetricia et gynecologica Scandinavica 2011;**90**(12):1317-24.
36. Khoshnood B, Lee K-s, Wall S, et al. Short Interpregnancy Intervals and the Risk of Adverse Birth Outcomes among 5 Racial/Ethnic Groups in the US. American Journal of Epidemiology 1998;**148**(8):798-805.
37. Blickstein I. Motherhood at or beyond the edge of reproductive age. International journal of fertility and women's medicine 2002;**48**(1):17-24.
38. Hilder L, Zhichao Z, Parker M, et al. Australia's mothers and babies 2012. Perinatal statistics series no. 30. Cat. no. PER 69. AIHW National Perinatal Epidemiology and Statistics Unit, Canberra 2014.
39. Matthews TJ, Hamilton BE. First births to older women continue to rise. NCHS data brief 2014(152):1-8.
40. Usta IM, Nassar AH. Advanced maternal age. Part I: obstetric complications. American journal of perinatology 2008;**25**(08):521-34.
41. Huang L, Sauve R, Birkett N, et al. Maternal age and risk of stillbirth: a systematic review. Canadian Medical Association Journal 2008;**178**(2):165-72.
42. Carolan M. Maternal age >45 years and maternal and perinatal outcomes: a review of the evidence. Midwifery 2013;**29**(5):479-89.

For peer review only - http://bmjopen.bmj.com/site/about/guidelines.xhtml

43. Bayrampour H, Heaman M. Advanced maternal age and the risk of cesarean birth: a systematic review. Birth 2010;**37**(3):219-26.
44. D'Agostino Jr RB. Propensity score methods for bias reduction in the comparison of a treatment to a non-randomized control group. Statistics in Medicine 1998;**17**(19):2265-81.
45. Cheslack-Postava K, Liu K, Bearman PS. Closely spaced pregnancies are associated with increased odds of autism in California sibling births. Pediatrics 2011;**127**(2):246-53.
46. Riley RD, Lambert PC, Abo-Zaid G. Meta-analysis of individual participant data: rationale, conduct, and reporting. BMJ 2010;**340**.
47. Egger M, Smith GD, Phillips AN. Meta-analysis: principles and procedures. BMJ : British Medical Journal 1997;**315**(7121):1533-37.
48. Higgins JPT, Thompson SG, Deeks JJ, et al. Measuring inconsistency in meta-analyses. BMJ : British Medical Journal 2003;**327**(7414):557-60.
49. Dildy, Jackson, Fowers, et al. Very advanced maternal age: Pregnancy after age 45. Am J Obstet Gynecol 1996;**175**(3, Part 1):668-74.

For peer review only

Figure 1: Effects of IPI on preterm birth (PTB) and low birth weight (LBW), with and without matching pregnancies to the same women, Western Australia, 1980-2010

Figures produced from statistics reported in Ball et al. 2014 [26]

For peer review only

Figure 2: Distribution of interpregnancy interval following a live birth, by country/state – 1980-2016

For peer review only

Table 1. Significant gaps in knowledge needed to inform recommendations for optimal interpregnancy interval recommendations in high-income countries.

Knowledge gap	Description
What are the IPI effects after eliminating confounding from between-women comparisons?	Few past studies have addressed confounding by matching pregnancies to the same women.[1]
Can IPI effects be observed later in childhood?	No past studies have investigated outcomes beyond the neonatal period, such as hospitalisation in early childhood.
What are the optimal IPIs for which risks are minimised?	Although knowledge of harmful IPIs is important, health is optimised by identification of optimal IPIs for which risks are minimised.[1]
What are the IPI effects for a wide range of outcomes of both the mother and child?	The vast majority of studies to date investigate a single endpoint for a single group (mother or child). IPI recommendations require a wide range of health endpoints for both mother and child.
What are the context-specific IPI recommendations?	Guideline IPIs are needed for sub-populations e.g., for women who attempt labour after caesarean birth to avoid uterine rupture,[29] and for women after stillbirth due to elevated risk of recurrence or faster maternal recovery. For women at advanced maternal age, delaying child birth even further due to IPI recommendations comes at the cost of competing risks of age-related morbidities.[49]

For peer review only

Table 2. Description of cohort and data sources used to identify a cohort of births in four high-income countries.

**Location**	**California, USA**	**Finland**	**Norway**	**New South Wales, Australia**	**Western Australia, Australia**
**Time Period**	1991-2010	1987-2017	1980-2016	1994-2016	1980-2015
**Data source**	Office of Statewide Health Planning and Development (OSHPD)	National Institute for Health and Welfare (THL), Medical Birth Register	Norwegian Institute of Public Health, Medical Birth Registry	NSW Perinatal Data Collection, NSW Ministry of Health	WA Midwives Data Collection, WA Department of Health
**Information available**	Maternal characteristics and health conditions; smoking and BMI (2007-2010); pregnancy and labour conditions, antenatal hospitalizations, information on delivery and birth outcomes; gestational age based on LMP estimate (obstetric estimate available for years 2007-2010)	Maternal characteristics and health conditions; smoking; pregnancy and labour complications; pregnancy history; details of antenatal care; information on delivery and birth outcomes; health of infant at discharge or 7 days	Maternal characteristics and health conditions; pregnancy and labour complications; medication use during pregnancy; birth outcomes; diagnoses of congenital abnormalities; parental occupation and smoking; births following assisted conception	Maternal characteristics and health conditions; smoking; pregnancy and labour complications; details of labour; birth outcomes; congenital anomalies, infant and child health outcomes	Maternal characteristics and health conditions; smoking; pregnancy and labour complications; details of labour; birth outcomes; congenital anomalies, infant and child health outcomes
**Scope of notified births**	All livebirths and stillbirths with Gestational length ≥20 weeks	All livebirths and stillbirths with gestational length ≥22 weeks or birthweight ≥500g	All pregnancies ending after week 12 from 2002 onwards (from week 16 from 1980-2001)	Gestational length ≥20 weeks or birthweight ≥400g	Gestational length ≥20 weeks or birthweight ≥400g
**Linkage methods**	Probabilistic linkage based on maternal descriptors	Deterministic linkage of mother based on Personal ID	Deterministic linkage of mother based on Personal ID	Probabilistic linkage based on maternal descriptors	Probabilistic linkage based on maternal descriptors
**Total births**	10.9 million	1.8 million	2.1 million	2.2 million	1.2 million
**Benchmark neonatal morbidity indicators**	10.5% PTB (LMP estimate)<br>6.4% LBW	5.8% PTB<br>4.3% LBW	5.7% PTB<br>3.4% LBW	5.4% PTB<br>4.4% LBW	5.3% PTB<br>5.3% LBW

For peer review only

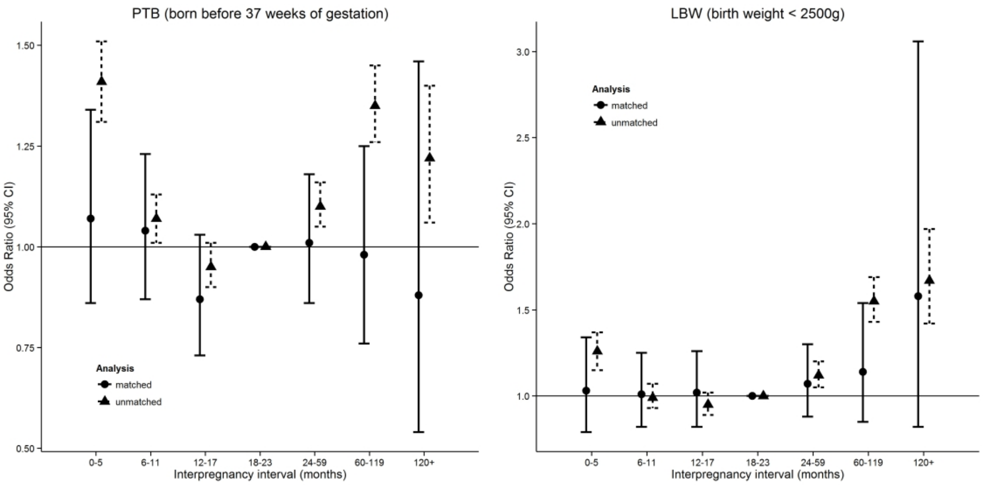

Figure 1: Effects of IPI on preterm birth (PTB) and low birth weight (LBW), with and without matching pregnancies to the same women, Western Australia, 1980-2010

Figures produced from statistics reported in Ball et al. 2014 [26]

355x177mm (300 x 300 DPI)

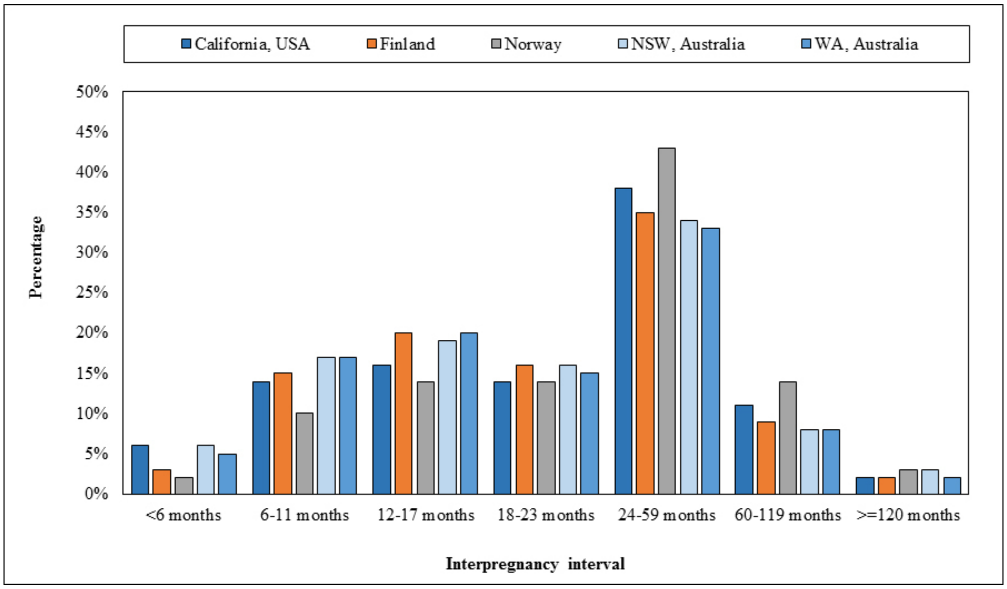

Figure 2: Distribution of interpregnancy interval following a live birth, by country/state – 1980-2016

76x44mm (300 x 300 DPI)

Table S1. Description of outcome ascertainment related to interpregnancy intervals in high-income countries and data sources for measuring outcomes.

Category	Measure/Data source	Description
Preterm birth	Length of gestation (completed weeks)	The quality of gestational length data has improved over time - for all cohorts, frequency of antenatal ultrasounds for pregnancy dating has increased, with corresponding declines in recording gestational lengths based on last menstrual period. PTB will be defined as all births <37 completed weeks gestation, and also sub-classified as idiopathic (spontaneous) and iatrogenic (medically indicated).
Fetal growth restriction based on SGA and low birthweight	Birth weight (grams)	LBW will be defined as weight <2500g. Birth weights are accurately measured by clinical personnel, with near-complete coverage. SGA will be defined as birth weight <10th centile (for gestational length and infant sex) of the birth weight distribution, by calendar year (which accounts for cohort effects), and by cohort (which accounts for differences between countries).
Congenital anomaly	WA, WA Register of Developmental Anomalies (WARDA)<br><br>NSW, Register of Congenital Conditions (RoCC)<br><br>Norway, Medical Birth Registry<br><br>Finland, Register of Congenital Malformations	The WARDA is a comprehensive database of all birth defects diagnosed up to 6 years of age, notifiable by legislation. A 5-year post-birth follow-up period will be used to identify birth defects. For NSW, the RoCC includes all infants diagnosed up to one year of age and only for a five-year period. The comprehensive case identification in WA will enable us to quantify non-identification of cases in the other cohorts, and weight the analyses accordingly.[50] For all cohorts combined, we estimate that >94% of cases with congenital anomaly will be identified.<br><br>In Finland, data on congenital anomalies are gathered from hospitals, health-care professionals and other national health registers. All cases recorded during pregnancy or infancy (<1 year) are included in the register. Statistical data are used for monitoring congenital anomalies nationally and regionally, and among other purposes, for planning prenatal screening, diagnostics and treatment of congenital anomalies as well as for conducting research on congenital anomalies. https://thl.fi/en/web/thlfi-en/statistics/information-on-statistics/register-descriptions/register-of-congenital-malformations.
Complications	Placental abruption, pPROM,	These databases have coverage of every birth within the state/country, and

For peer review only

of pregnancy and labour	labour dystocia, uterine rupture, pre-eclampsia, gestational hypertension, gestational diabetes<br><br>Midwives Data Collection (WA), Perinatal Data Collection (NSW), maternal antenatal hospitalisations (WA, NSW), birth certificates (California) and the Medical Birth Registers (Finland, Norway).	the complications of labour and pregnancy are recorded at birth. For California, ICD coded complications (ICD-9) of pregnancy and labour that resulted in antenatal hospitalisation are additionally included on the birth certificate records themselves, and will be used to supplement case identification. For WA, NSW and Finland, case identification will be supplemented with a search of the following primary and secondary diagnosis codes (ICD-9/10) of all hospital admissions between start of pregnancy and the discharge date of the birth admission of the mother. Placental abruption: 641.2, O45; labour dystocia: 662.0, O63.0; uterine rupture: 665.1, O71.0, O71.1; pre-eclampsia: 642.4, 642.5, 642.7, O14; gestational hypertension: 642.3, O13; pPROM: 658.1, 658.2 (+ gestational length <37wks), O42; gestational diabetes: 648.8, O24.4
Infant and child health outcomes	Hospital admissions (WA, NSW, Finland)	Hospital contacts will identified from hospital admissions (WA, NSW, Finland) with 3-year follow-up (birth-age 3 years).
Perinatal, infant and maternal mortality	Death registrations (WA, NSW, Norway, Finland) and death certificates (California).	These data sources record every death registered in the state/country. The date of death will be utilised to identify stillbirths (week 20 gestation – birth), perinatal mortality (stillbirths + neonatal mortality), infant mortality (28 days – age 1 year) early childhood mortality (age 1 year to age 3 years), and maternal mortality (pregnancy period – 42 days postpartum). Accidental death, homicide, and other external causes will be identified with cause of death codes and subsequently excluded. Hospital contacts will identified from hospital admissions (WA, NSW, Finland) with 3-year follow-up (birth-age 3 years) and investigated as binary outcomes (>0; >10th,25th,50^{th}centiles of frequency).

For peer review only

Table S2: Factors affecting pregnancy intervals identified by our Healthy Pregnancy Consumer Reference Group.

Psychological factors	*Replace* a pregnancy loss; compensate for a previous adverse birth outcome; psychological preparation – fear of pregnancy loss or adverse birth outcome in the previous pregnancy; sexual activity is a reminder of pregnancy loss, disability or other adverse birth outcome experienced; relationship stability – partner unsupportive of increasing family size, relationship difficulties, no partner; postnatal depression after the first birth; depression, anxiety, stress, flare of an existing mental health problem or difficulty managing medication while pregnant; experience of a stress-event (death of a loved one, moving home, job loss, divorce/separation); reproductive coercion/abuse.
**Healthcare provision**	Lack of information from healthcare professionals as to clinical recommendations on birth spacing, and their associated risks; encouragement by obstetricians to try again for a child soon after pregnancy loss; information GPs that breastfeeding prevents pregnancy without provision of guidance; no information provided by specialists on risks associated with timing of pregnancy.
**Lifestyle factors/choices**	*Not miss out* - experience what they had planned but could not experience in the first pregnancy; advanced parental age; sex balance desired in family – trying for a girl/boy; household preparation – residential mobility, physical space in the home; ability to manage the day-to-day care of the current child or children – with/without first child morbidities; maternal career aspirations; physical preparation to improve fitness and health; desire to replicate/avoid age gaps in family members; change in partner; multiple gestation in first pregnancy.
**Support and social network**	Availability of support from family – childcare; living in a region distant from extended family; couple living/working in different regions from each other; increase in family size of friends and family; interference from parents/in-laws in the marriage or parenting.
**Contraception sexual activity Fecundity**	Lack of contraceptive use or proper contraceptive use; lack of knowledge regarding pregnancy planning e.g., breast feeding required lactational amenorrhea; failed contraception; higher levels of sexual activity; couple living/working away or work schedules clashing (e.g., shift work); difficulty falling pregnant; reproductive treatment; failed treatment; delays in treatment.
**Financial factors**	Higher paternal income; lower paternal income or reliance on maternal income; cost of contraception; financial preparation – savings, debt; cost of fertility treatment; cost of childcare – more than one child in childcare at the same time is financially prohibitive; paid maternity leave requirements e.g., returning to work for at least 1 year to become eligible.
**Morbidity Healing**	Major morbidities including heart disease, malignant neoplasm; known recurrent pregnancy issues (e.g., hyperemesis gravidarum); healing time required after illness or the previous pregnancy; fear of effects of medication on the fetus; hormonal therapy (progesterone, HCG).

For peer review only

BMJ Open

Developing evidence-based recommendations for optimal interpregnancy intervals in high-income countries: protocol for an international cohort study

Journal:	*BMJ Open*
Manuscript ID	bmjopen-2018-027941.R1
Article Type:	Protocol
Date Submitted by the Author:	23-Nov-2018
Complete List of Authors:	Marinovich, M Luke; Curtin University, School of Public Health<br>Regan, Annette; Texas A&M University, School of Public Health<br>Gissler, Mika; THL National Institute for Health and Welfare, Information<br>Magnus, Maria; University of Bristol, MRC Integrative Epidemiology Unit; Norwegian Institute of Public Health, Centre for Fertility and Health (CeFH)<br>Håberg, Siri; Norwegian Institute of Public Health,<br>Padula, Amy; University of California San Francisco Department of Obstetrics Gynecology and Reproductive Sciences,<br>Mayo, Jonathan; Stanford University, Department of Pediatrics<br>Shaw, Gary M.; Stanford University, Department of Pediatrics<br>Ball, Stephen; Curtin University, Prehospital, Resuscitation and Emergency Care Research Unit (PRECRU), School of Nursing, Midwifery and Paramedicine<br>malacova, eva; Curtin University, School of Public Health<br>Gebremedhin, Amanuel; Curtin University, School of Public Health<br>Nassar, Natasha; University of Sydney, Menzies Centre for Health Policy, School of Public Health<br>Marston, Cicely; London School of Hygiene and Tropical Medicine, Faculty of Public Health and Policy<br>de Klerk, Nick; University of Western Australia, Telethon Kids Institute<br>Betran, Ana Pilar; World Health Organization, Department of Reproductive Health and Research<br>Pereira, G.F; Curtin University, School of Public Health
**Primary Subject Heading**:	Obstetrics and gynaecology
Secondary Subject Heading:	Epidemiology, Paediatrics, Public health, Health policy
Keywords:	pregnancy, birth intervals, family planning, siblings, preterm birth, fetal growth restriction

SCHOLARONE™
Manuscripts

Developing evidence-based recommendations for optimal interpregnancy intervals in high-income countries: protocol for an international cohort study

M Luke Marinovich,[1] Annette K Regan,[2] Mika Gissler,[3,4] Maria C Magnus,[5,6,7] Siri Håberg,[7] Amy M Padula,[8] Jonathan A Mayo,[9] Gary M Shaw,[9] Stephen Ball,[10] Eva Malacova,[1] Amanuel T Gebremedhin,[1] Natasha Nassar,[11] Cicely Marston,[12] Nick de Klerk,[13] Ana Pilar Betrán,[14] Gavin Pereira[1]

Author affiliations:

[1] School of Public Health, Curtin University, Perth, Western Australia, Australia

[2] School of Public Health,Texas A&M University, College Station, Texas, USA

[3] Information Services Department, National Institute for Health and Welfare, Helsinki, Finland

[4] Department of Neurobiology, Care Sciences and Society, Karolinska Institute, Stockholm, Sweden

[5] MRC Integrative Epidemiology Unit at the University of Bristol, Bristol, United Kingdom

[6] Population Health Sciences, Bristol Medical School, Bristol, United Kingdom

[7] Centre for Fertility and Health (CeFH), Norwegian Institute of Public Health, Oslo, Norway

[8] Department of Obstetrics, Gynecology and Reproductive Sciences, University of California, San Francisco, USA

[9] Department of Pediatrics, Stanford University, Stanford, USA

[10] School of Nursing, Midwifery and Paramedicine, Curtin University, Perth, Western Australia, Australia

[11] Menzies Centre for Health Policy, School of Public Health, University of Sydney, Sydney, New South Wales, Australia

[12] Faculty of Public Health and Policy, London School of Hygiene and Tropical Medicine, London, UK

[13] Telethon Kids Institute, University of Western Australia, Subiaco, Western Australia, Australia

[14] UNDP/UNFPA/UNICEF/WHO/World Bank Special Programme of Research, Development and Research Training in Human Reproduction, Department of Reproductive Health and Research, World Health Organization, Geneva, Switzerland

Corresponding author:

Dr ML Marinovich, Research Fellow
School of Public Health, Curtin University
GPO Box U1987
Perth Western Australia 6845
Email: Luke.Marinovich@curtin.edu.au
Phone: +61 8 9266 4006

Protocol version: 1.0 (November 2018)

Word count: 2735

For peer review only - http://bmjopen.bmj.com/site/about/guidelines.xhtml

ABSTRACT

Introduction: Short interpregnancy interval (IPI) has been linked to adverse pregnancy outcomes. The World Health Organization (WHO) recommends waiting at least two years after a live birth and six months after miscarriage or induced termination before conception of another pregnancy. The evidence underpinning these recommendations largely relies on data from low and middle-income countries. Furthermore, recent epidemiological investigations have suggested that these studies may over-estimate the effects of IPI due to residual confounding. Future investigations of IPI effects in high-income countries drawing from large, population-based data sources are needed to inform IPI recommendations. We aim to assess the impact of IPIs on maternal and child health outcomes in high-income countries.

Methods and Analysis: This international longitudinal retrospective cohort study will include more than 18 million pregnancies, making it the largest study to investigate IPI in high-income countries. Population-based data from Australia, Finland, Norway, and US will be utilised. Birth records in each country will be used to identify consecutive pregnancies. Exact dates of birth and clinical best estimates of gestational length will be used to estimate IPI. Administrative birth and health data sources with >99% coverage in each country will be used to identify maternal sociodemographics, pregnancy complications, details of labour and delivery, birth and child health information. We will use matched and unmatched regression models to investigate the impact of IPI on maternal and infant outcomes, and conduct meta-analysis to pool results across countries.

Ethics and Dissemination: Ethics boards in at participating sites approved this research (approval was not required in Finland). Findings will be published in peer-reviewed journals and presented at international conferences, and will inform recommendations for optimal IPI in high-income countries. Findings will provide important information for women and

For peer review only - http://bmjopen.bmj.com/site/about/guidelines.xhtml

families planning future pregnancies and for clinicians providing prenatal care and giving guidance on family planning.

Keywords: pregnancy; birth intervals; family planning; siblings; preterm birth; fetal growth restriction

For peer review only

ARTICLE SUMMARY

Strengths and limitations of this study

- With data from four countries and over 18 million pregnancies, this will be the largest cohort study to investigate IPI in high-income countries.
- The size of this cohort will allow strict control for confounding through matching multiple pregnancies within women, and permit the investigation of IPI effects among sub-populations.
- Collaboration between countries and the use of individual-level data will minimise methodological heterogeneity.
- Data items such as socioeconomic status vary between countries in their completeness and their method of measurement, limiting the extent to which such variables may be incorporated as covariates.
- Data on early pregnancy loss are poorly or inconsistently captured in routine data collection, which may result in misclassification of IPI.

For peer review only

INTRODUCTION

Interpregnancy interval (IPI), or the time from birth to conception of the next pregnancy, has been identified as a potentially modifiable risk factor linked to adverse perinatal outcomes. Currently, the World Health Organization (WHO) recommends that women wait at least two years after a live birth and six months after early pregnancy loss before conceiving again to reduce the risk of adverse maternal and perinatal outcomes.[1] This recommendation is based on observational studies, mostly from low and middle-income countries, that have demonstrated associations between short IPI and adverse pregnancy outcomes,[2] most notably maternal mortality,[3] small for gestational age (SGA), term low birth weight (LBW),[4] preterm pre-labour rupture of membranes (pPROM),[5,6] preterm birth (PTB)[4,7,8] and birth defects.[9,10] Furthermore, long IPI has been shown to be associated with higher risk of outcomes such as fetal death,[11] LBW, PTB, SGA,[12] and pre-eclampsia[11,13].

Several theories have been proposed to explain these associations. Short IPIs may leave insufficient time to recover from maternal nutrient deficits, which can lead to fetal-maternal competition for essential nutrients.[14] This can be exacerbated by lactation[15] and malnourishment, in both high and low-income countries.[16,17] Short IPIs might also leave insufficient recovery time from inflammatory processes from the previous pregnancy that extend into the next pregnancy.[2] Long IPIs may result in the loss of adaptive benefits to the mother from a previous birth, resulting in a return to a state equivalent to primigravida.[17] An alternative hypothesis is that the observed associations between IPI and adverse pregnancy outcomes can be explained, at least partially, by systematic bias.[18] A spurious association would result if time to conception were independently associated with other factors causally linked to adverse pregnancy outcomes in the subsequent pregnancy.[19,20] For example, patterns in IPI are associated with maternal age, socioeconomic status (SES),

For peer review only - http://bmjopen.bmj.com/site/about/guidelines.xhtml

breastfeeding, and other antenatal, postnatal, or postpartum practices.[21,22] Furthermore, many factors that can confound the associations between IPI and adverse pregnancy outcomes, such as psychosocial determinants of health, are difficult to measure. Undoubtedly, the greatest challenge for observational studies is to comprehensively account for such confounding factors. High quality studies have adjusted for potential confounders, but statistical adjustment is rarely complete. Residual unmeasured confounding can lead to bias[23] of the observed effect of IPI on adverse pregnancy outcomes. Importantly, as the mechanisms by which IPI affects specific pregnancy outcomes are not well-understood, direct adjustment cannot be made for confounders that remain unknown. Under this scenario, the current state of knowledge on the effects of IPI is clinically unreliable.

In recent years, studies have attempted to address the issue of bias from unmeasured confounding through a matched longitudinal study design.[24-26] Longitudinal study designs which match pregnancies to the same women[26,27] can better overcome confounding introduced by unknown, unmeasured and inaccurately measured factors that tend to vary between mothers but remain similar between pregnancies.[24,25,28] Findings from these studies suggest the effects of IPI reported from unmatched models are over-estimated. Considerable reductions in the U-shape effect of IPI are observed after matching pregnancies to the same women (Figure 1),[26] indicating support for the systematic bias hypothesis. These results provide a persuasive argument that some of the IPI effect observed in past studies may be attributable to confounders that vary between women (e.g., heritable/genetic, socioeconomic, recurrent health-related behaviour, unknown/unmeasured), but can be effectively controlled under a longitudinal matched design.

Although these results cast doubt over the adverse effects of IPI, the existence of adverse associations with perinatal outcomes and the optimal IPI period remains unclear due to smaller sample sizes for matched analyses. Confidence intervals around estimates are

For peer review only - http://bmjopen.bmj.com/site/about/guidelines.xhtml

typically wider for matched analyses which rely on women having at least two IPIs during the study period, particularly for the effects of longer IPI (>60 months). In addition, the selection of women with more than two pregnancies in matched analyses limits generalisability to all women. Several other gaps remain in our knowledge around IPI effects (Table 1). First, the majority of studies have so far been restricted to low and middle-income countries. For example, the current WHO recommendation for IPI following early pregnancy loss are based on the findings from a single, albeit large, study in Latin America,[4] and it remains unclear as to whether the findings are relevant to high-income countries. Second, some of the most pertinent questions for women in high-income countries, such as those related to obstetric context, have not yet been addressed. Additional data from high-income countries and more robust results are needed.

Study aims

The primary aim of this study is to evaluate the impact of IPI on pregnancy outcomes in high-income countries. Secondary aims are to:

1. **<u>Evaluate the impact of IPI based on obstetric context</u>:** IPI consequences may differ for women based on obstetric history. For example, optimal IPI may vary for women who previously gave birth by caesarean section due to risk of uterine rupture after insufficient time for the uterine scar to heal.[29] The effect of IPI may also vary depending on gestational age of the previous birth, since longer gestation could be expected to prolong the maternal nutrient deficits and inflammatory processes implicated in poorer outcomes for the subsequent birth, particularly with short IPI. Furthermore, recommendations of IPI after stillbirth require specific attention[30]. Pregnancy intervals for women following a stillbirth or neonatal loss are approximately one year shorter than intervals for women without a loss.[31,32] It has been speculated that this is due to parents wishing to counter the emotional loss of

For peer review only - http://bmjopen.bmj.com/site/about/guidelines.xhtml

their infant or to minimise the delay in having a baby.[33] However, a survey of obstetricians regarding the timing of pregnancy following pregnancy loss found approximately two thirds of obstetricians endorse women waiting less than 6 months after stillbirth before trying to conceive a new pregnancy.[34] This is incongruous with the WHO IPI recommendations that women wait at least 6 months after pregnancy loss.

2. **Evaluate the impact of IPI based on socio-demographic context:** The IPI recommendations generated by this study may be most beneficial for those populations for whom we know short IPI and high parity (large family size) is prevalent. Within high-income countries, both family size and the IPI distribution can vary markedly according to race/ethnicity and SES.[35,36] In this study, we will investigate the effects of IPI among women from different socioeconomic and racial/ethnic groups in multiple high-income countries.

3. **Evaluate the potential influence of maternal age on the effect of IPI on pregnancy outcomes:** In high income countries, age at childbirth overall is increasing, with later age at first birth.[37-39] Systematic reviews conclude that increased maternal age is associated with greater risk of a wide range of adverse obstetric outcomes,[40] including perinatal mortality,[41] PTB and LBW,[42] and pregnancy intervention, particularly caesarean section.[43] In this study, we will test the hypothesis that there is an interaction between increased maternal age and IPI on the risk of adverse pregnancy outcomes. For each year of maternal age (i.e. maternal age at first observed birth in the study period) and for different IPIs, we will calculate the risk of adverse pregnancy outcomes. This will provide valuable information on

For peer review only - http://bmjopen.bmj.com/site/about/guidelines.xhtml

any combined risks of maternal age and IPI for older women conceiving their second (or later) child.

METHODS AND ANALYSIS

Study design and population

We will conduct a longitudinal retrospective cohort study on the effects of IPI on maternal and child health outcomes using individual-level records from with near complete coverage of all births (>99%) in Australia (Western Australia [WA] and New South Wales [NSW]), Finland, Norway, and the United States (US; California) (Table 2).

We will conduct analyses using unmatched (all women) and matched approaches. By matching pregnancies to the same women, we will account for individual-level confounders (known and unknown) that remain stable between pregnancies. The effect of IPI from matched and unmatched analyses will be compared. The study's population will consist of more than 18 million births, which will be the largest IPI study for high-income countries. The size of this cohort offers several advantages, as it allows us to: a) investigate IPI effects among sub-populations, and b) ensure the best possible control for confounding. Where within-mother matching is not possible (e.g. when studying effect-modification, sample size in individual data categories becomes reduced), we will match on propensity scores.[44]

Inclusion criteria

Women with two or more consecutive pregnancies will be included in the study. The target population for inference includes women with >1 birth during their life course. IPI is not relevant for women who have only one birth. Given that matching pregnancies to the same women requires >1 pregnancy interval per woman, the cohort for matched analyses will be restricted to women who have 2 or more births during the study period. The 20 to 30-year

For peer review only - http://bmjopen.bmj.com/site/about/guidelines.xhtml

study period ensures sufficient follow-up to observe final family size. Birth status (live born vs stillborn), plurality (singleton vs multiple) and maternal age will inform inclusion criteria for specific hypotheses.

Definition of IPI

IPI will be defined as the length of time between the start of the index pregnancy (birth date minus gestational length) and the birth date of the preceding pregnancy. IPI will be classified using integer months and categorised as: 0-5, 6-11, 12-17, 18-23, 24-59, 60-119, and ≥120 months, with 18-23 months used as the referent group.[26] These intervals share endpoints consistent with recommendations of past studies and WHO recommendations. In addition, IPI will be considered as a continuous variable for assessment of optimal IPI.

IPI-relevant health outcomes

Based on a review of the current literature, we identified a set of clinically significant IPI-relevant endpoints, contexts and competing risks that can be investigated accurately and reliably (Table S1). We classified the *primary* IPI-relevant endpoints into the following two categories: preterm birth (PTB); and fetal growth restriction based on the definitions of small for gestational age (SGA) and low birthweight (LBW). Additional endpoints include congenital anomaly; complications of pregnancy and labour; infant and child health outcomes; and perinatal, infant and maternal mortality.

Data sources

Cohorts will be assembled from linked birth cohort files in California with Office of Statewide Health Planning and Development maternal and infant hospital discharge data (USA: 1991-2010); the National Institute for Health and Welfare (THL), Medical Birth Register (Finland: 1987-2017); the Norwegian Institute of Public Health, Medical Birth Registry (Norway: 1980-2015); the NSW Perinatal Data Collection, NSW Ministry of Health

For peer review only - http://bmjopen.bmj.com/site/about/guidelines.xhtml

(Australia: 1994-2016); and the WA Midwives Data Collection, WA Department of Health (Australia: 1980-2015) (Table 2). Coverage is >99% of births for each country/jurisdiction. IPIs for each data source will be derived (Figure 2), and maternal characteristics and health conditions, pregnancy complications, details of labour and childbirth, birth outcomes and mortality will be identified (Table S1). Some sites will have access to additional linked hospital and child development data, offering the opportunity to explore the impact on additional maternal and child health outcome measures, including congenital anomalies (WA, NSW, Norway, Finland) and hospital admissions (WA, NSW, Finland).

Data de-identification and secure storage

De-identified data from California and Finland will be retained and held securely at those sites; de-identified data from Norway, WA and NSW will be transferred to and stored at the School of Public Health, Curtin University. Project data will be electronically stored on a secure server, which is backed up daily to prevent any unintentional data loss. The research environment includes a variety of security controls to restrict unauthorised access – these include access controls, role-based delegations, encryption, firewalls, and physical access restrictions (authorised access to server rooms and research offices is restricted by key). Automatic screen locking will occur on electronic devices after five minutes of inactivity. Data will not be stored or used in public terminals. No paper-based or portable electronic media storage of data will take place.

Statistical methods

Matched and unmatched logistic regression will be used to investigate all maternal and child health outcomes (Table S1). For primary analyses, mother ID will be used as the matching variable to identify strata. This method ensures that the results will be based entirely on within-women (not between-women) comparisons, minimising the need for additional

For peer review only - http://bmjopen.bmj.com/site/about/guidelines.xhtml

adjustment and is the standard statistical method for matched studies. We will explicitly adjust for factors of the index pregnancy that do/can change between pregnancies. Specifically, we will adjust for maternal age (categorical variable: 14-19, 20-24, 25-29, 30-34, 35-39, and ≥40 years), parity (categorical), and conception year (non-linear spline). Due to differences between countries in the completeness and method of measurement of SES (or maternal education as a proxy), adjustment for SES will not be possible when data are pooled across countries, but may be undertaken when analyses are conducted using single-country datasets.

Matched analyses will be undertaken to address secondary aims, where possible. In circumstances where within-mother matching is not feasible or not possible (e.g. sub-populations with small sample sizes which would be further restricted by requiring more than 2 consecutive pregnancies per mother), unmatched models or alternative matching methods will be used (e.g. propensity score stratification; sibling-matched methods using a single IPI).[8,44,45]

Analyses will first be applied to each site's data individually. The analytic approach and statistical code used for data sets held at Curtin University (WA, NSW, Norway) will be sent to the other sites (California, Finland) to replicate analyses, allowing standardisation and minimising methodological heterogeneity between countries[46]. Meta-analysis[47] will be used to combine adjusted odds ratios (ORs) obtained from each data source into a single pooled estimate generalisable to high-income countries. Heterogeneity between countries will be investigated with the I^2 statistic.[48]

Patient and public involvement

A reference group of consumer health representatives (*Healthy Pregnancies Reference Group)* has been established, and will meet twice-yearly to provide a community perspective

For peer review only

For peer review only - http://bmjopen.bmj.com/site/about/guidelines.xhtml

on this research. The reference group will provide advice regarding the aims of the research; language, including lay summaries; links between consumers, the community, and the researchers; and advocacy on behalf of consumers and the community. The reference group will also contribute consumer perspectives on potential utilisation of the research findings, such as the identification of factors that may influence IPI (Table S2), with the intention to inform further primary research in this area.

ETHICS AND DISSEMINATION

Human research ethics committee approval

This research has ethics approval from the Department of Health Western Australia Human Research Ethics Committee; Curtin University Human Research Ethics Committee; NSW Population and Health Services Research Ethics Committee; Stanford University Institutional Review Board; and the Norwegian Regional Committees for Medical and Health Research Ethics. Each committee provided a waiver of consent for participants. For Finland, ethical approval was not required.

Intended publications and research dissemination

Datasets generated and/or analysed during the current study are not publicly available due to data confidentiality agreements with data custodians. Results generated by the research will be made publicly available at the summary level. Standalone manuscripts addressing primary and secondary aims will be published in peer-reviewed journals. Results will also be presented at relevant perinatal and epidemiological conferences. Findings will be used to develop guidelines for IPI as a potentially modifiable risk factor linked to adverse perinatal outcomes, specifically for women in high-income countries.

For peer review only - http://bmjopen.bmj.com/site/about/guidelines.xhtml

DISCUSSION

This study will inform new IPI recommendations for women in high-income countries. Recommendations will be specific to obstetric and socioeconomic context. By combining record-linked perinatal data from multiple countries – Australia, Finland, Norway, and the US – this study will achieve the largest sample of high-quality health data in high-income countries. Novel methodology, matching pregnancies to the same women, will achieve the best control for confounding of any study to date.

This international cohort approach offers several advantages. Large sample sizes are required to undertake matched analyses with adequate statistical power to detect clinically meaningful differences between IPIs. Prospective studies of this size are infeasible. This study can only be achieved using linked perinatal data within an international collaboration. Collaboration to standardise methods between countries will also minimise methodological heterogeneity in pooled analyses. Furthermore, this research has the potential to be extended by broadening the collaboration to incorporate additional data sets. The minimum data requirements for contributing to this project are: 1) unique identifier for mothers to link multiple pregnancies; 2) mother's date of birth; 3) child's date of birth; 4) child's gestational length; and 5) other birth and early childhood outcomes as described in Table S1. Researchers interested in joining this collaboration are encouraged to contact us through the corresponding author.

This information is essential for supporting family planning, both at the patient and provider level. Evidence-based recommendations for IPI for women in high-income countries are useful for assisting families when planning future pregnancies and for clinicians when providing intra-pregnancy counselling.

For peer review only

List of abbreviations

IPI, interpregnancy interval

LBW, low birthweight

NSW, New South Wales

OR, odds ratio

pPROM, preterm premature rupture of membranes

PTB, preterm birth

SES, socioeconomic status

SGA, small-for-gestational age

US, United States

WA, Western Australia

WHO, World Health Organization

DECLARATIONS

Ethics

This study was approved by the Department of Health Western Australia Human Research Ethics Committee; Curtin University Human Research Ethics Committee; NSW Population and Health Services Research Ethics Committee; Stanford University Institutional Review Board; and the Norwegian Regional Committees for Medical and Health Research Ethics. Each committee provided a waiver of consent for participants. For Finland, ethical approval was not required.

Consent for publication

The authors confirm this work is original and has not been published elsewhere, in part or in whole.

Competing interests

The authors have no competing interests to declare.

Funding

For peer review only - http://bmjopen.bmj.com/site/about/guidelines.xhtml

For peer review only

This work was supported by funding from the National Health and Medical Research Council, including an Early Career Fellowship [#GNT1138425 to AR], Senior Research Fellowship [# 1117105 to HL], Career Development Fellowship [#APP1067066 to NN] and project grants [#1099655 to GP, NN, SJB; #APP1141510 to AR, GP]. MCM works at the MRC Integrative Epidemiology Unit at the University of Bristol which receives infrastructure funding from UK Medical Research Council (MC/UU/12013/5), and she is also supported by the following fellowship from the UK Medical Research Council (MR/M009351/1). This work was partly supported by the Research Council of Norway through its Centres of Excellence funding scheme, project number 262700. The authors have no potential conflicts of interests to disclose.

Author contributions

GP, SB, AR and MLM conceived the idea, planned and designed the study protocol. GP, SB, AR, MLM, MG, MCM, SH, AMP, JAM, GMS, EM, ATG, NN, CM, NdK and AP contributed to the development of the protocol, study design and methods. MLM and AR wrote the first draft. MG, MCM, SH, AMP, JAM, GMS, SB, EM, ATG, NN, CM, NdK, AP and GP critically revised the draft for important intellectual content. All authors have approved the final written manuscript.

Acknowledgements

The authors would like to thank the Linkage and Client Services Teams at the Data Linkage Branch (Department of Health Western Australia) as well as the Data Custodian for the Midwives Notification System. The authors would also like to thank members of the Healthy Pregnancies Consumer Reference Group who provided community input and guidance in this research.

For peer review only - http://bmjopen.bmj.com/site/about/guidelines.xhtml

References

1. Marston C. Report of a WHO Technical Consultation on Birth Spacing Geneva Switzerland 13-15 June 2005: World Health Organization, 2007.
2. Shachar BZ, Lyell DJ. Interpregnancy interval and obstetrical complications. Obstetrical & gynecological survey 2012;**67**(9):584-96.
3. Conde-Agudelo A, Belizán JM. Maternal morbidity and mortality associated with interpregnancy interval: cross sectional study. Bmj 2000;**321**(7271):1255-59.
4. Conde-Agudelo A, Belizán JM, Norton MH, et al. Effect of the Interpregnancy Interval on Perinatal Outcomes in Latin America. Obstetrics & Gynecology 2005;**106**(2):359-66 10.1097/01.AOG.0000171118.79529.a3.
5. Getahun D, Strickland D, Ananth CV, et al. Recurrence of preterm premature rupture of membranes in relation to interval between pregnancies. Am J Obstet Gynecol 2010;**202**(6):570. e1-70. e6.
6. Razzaque A, Da Vanzo J, Rahman M, et al. Pregnancy spacing and maternal morbidity in Matlab, Bangladesh. International Journal of Gynecology & Obstetrics 2005;**89**:S41-S49.
7. Smith GCS, Pell JP, Dobbie R. *Interpregnancy interval and risk of preterm birth and neonatal death: retrospective cohort study*, 2003.
8. Regan AK, Ball SJ, Warren JL, et al. A Population-based Matched Sibling Analysis Estimating the Association between First Interpregnancy Interval and Birth Outcomes. American journal of epidemiology 2018.
9. Grisaru-Granovsky S, Gordon E-S, Haklai Z, et al. Effect of interpregnancy interval on adverse perinatal outcomes—a national study. Contraception 2009;**80**(6):512-18.
10. Kwon S, Lazo-Escalante M, Villaran M, et al. Relationship between interpregnancy interval and birth defects in Washington State. Journal of Perinatology 2011;**32**(1):45-50.
11. Mignini LE, Carroli G, Betran AP, et al. Interpregnancy interval and perinatal outcomes across Latin America from 1990 to 2009: a large multi-country study. BJOG : an international journal of obstetrics and gynaecology 2016;**123**(5):730-37.
12. Conde-Agudelo A, Rosas-Bermúdez A, Kafury-Goeta AC. Birth spacing and risk of adverse perinatal outcomes: a meta-analysis. Jama 2006;**295**(15):1809-23.
13. Cormick G, Betrán AP, Ciapponi A, et al. Inter-pregnancy interval and risk of recurrent pre-eclampsia: systematic review and meta-analysis. Reproductive Health 2016;**13**:83.
14. Winkvist A, Rasmussen KM, Habicht J-P. A new definition of maternal depletion syndrome. American journal of public health 1992;**82**(5):691-94.
15. Adair LS, Popkin BM. Prolonged lactation contributes to depletion of maternal energy reserves in Filipino women. The Journal of nutrition 1992;**122**(8):1643-55.
16. Adam I, Ismail MH, Nasr AM, et al. Low birth weight, preterm birth and short interpregnancy interval in Sudan. Journal of Maternal-Fetal and Neonatal Medicine 2009;**22**(11):1068-71.
17. Zhu B-P, Rolfs RT, Nangle BE, et al. Effect of the interval between pregnancies on perinatal outcomes. New England journal of medicine 1999;**340**(8):589-94.
18. Miller JE. Birth intervals and perinatal health: an investigation of three hypotheses. Family planning perspectives 1991:62-70.
19. Sholapurkar S. Is there an ideal interpregnancy interval after a live birth, miscarriage or other adverse pregnancy outcomes? Journal of Obstetrics & Gynaecology 2010;**30**(2):107-10.
20. Thom DH, Nelson LM, Vaughan TL. Spontaneous abortion and subsequent adverse birth outcomes. Am J Obstet Gynecol 1992;**166**(1):111-16.
21. Flacking R, Nyqvist KH, Ewald U. Effects of socioeconomic status on breastfeeding duration in mothers of preterm and term infants. The European Journal of Public Health 2007;**17**(6):579-84.
22. Blakely T, Hunt D, Woodward A. Confounding by socioeconomic position remains after adjusting for neighbourhood deprivation: an example using smoking and mortality. Journal of Epidemiology and Community Health 2004;**58**(12):1030-31.

For peer review only - http://bmjopen.bmj.com/site/about/guidelines.xhtml

23. Kaufman JS, Cooper RS, McGee DL. Socioeconomic Status and Health in Blacks and Whites: The Problem of Residual Confounding and the Resiliency of Race. Epidemiology 1997;**8**(6):621-28.
24. Shachar BZ, Mayo JA, Lyell DJ, et al. Interpregnancy interval after live birth or pregnancy termination and estimated risk of preterm birth: a retrospective cohort study. BJOG : an international journal of obstetrics and gynaecology 2016;**123**(12):2009-17.
25. Hanley GE, Hutcheon JA, Kinniburgh BA, et al. Interpregnancy Interval and Adverse Pregnancy Outcomes: An Analysis of Successive Pregnancies. Obstetrics and gynecology 2017;**129**(3):408-15.
26. Ball SJ, Pereira G, Jacoby P, et al. Re-evaluation of link between interpregnancy interval and adverse birth outcomes: retrospective cohort study matching two intervals per mother. BMJ 2014;**349**:g4333.
27. Pereira G, Bell ML, Lee HJ, et al. Sources of Fine Particulate Matter and Risk of Preterm Birth in Connecticut, 2000–2006: A Longitudinal Study. Environ Health Perspect 2014.
28. Susser E, Eide MG, Begg M. Invited Commentary: The Use of Sibship Studies to Detect Familial Confounding. American journal of epidemiology 2010;**172**(5):537-39.
29. Conde-Agudelo A, Rosas-Bermúdez A, Kafury-Goeta AC. Effects of birth spacing on maternal health: a systematic review. Am J Obstet Gynecol 2007;**196**(4):297-308.
30. Regan AK, Gissler M, Magnus MC, et al. Adverse birth outcomes associated with interpregnancy interval among women with a previous stillbirth: an international cohort study. Lancet in press.
31. August EM, Salihu HM, Weldeselasse H, et al. Infant mortality and subsequent risk of stillbirth: a retrospective cohort study. BJOG : an international journal of obstetrics and gynaecology 2011;**118**(13):1636-45.
32. Black M, Shetty A, Bhattacharya S. Obstetric outcomes subsequent to intrauterine death in the first pregnancy. BJOG : an international journal of obstetrics and gynaecology 2008;**115**(2):269-74.
33. Rowland Hogue CJ, Silver RM. Racial and ethnic disparities in United States: stillbirth rates: trends, risk factors, and research needs. Seminars in perinatology 2011;**35**(4):221-33.
34. Gold KJ, Leon I, Chames MC. National survey of obstetrician attitudes about timing the subsequent pregnancy after perinatal death. Am J Obstet Gynecol 2010;**202**(4):357.e1-57.e6.
35. Hogue CJ, Menon R, Dunlop AL, et al. Racial disparities in preterm birth rates and short inter-pregnancy interval: an overview. Acta obstetricia et gynecologica Scandinavica 2011;**90**(12):1317-24.
36. Khoshnood B, Lee K-s, Wall S, et al. Short Interpregnancy Intervals and the Risk of Adverse Birth Outcomes among 5 Racial/Ethnic Groups in the US. American Journal of Epidemiology 1998;**148**(8):798-805.
37. Blickstein I. Motherhood at or beyond the edge of reproductive age. International journal of fertility and women's medicine 2002;**48**(1):17-24.
38. Hilder L, Zhichao Z, Parker M, et al. Australia's mothers and babies 2012. Perinatal statistics series no. 30. Cat. no. PER 69. AIHW National Perinatal Epidemiology and Statistics Unit, Canberra 2014.
39. Matthews TJ, Hamilton BE. First births to older women continue to rise. NCHS data brief 2014(152):1-8.
40. Usta IM, Nassar AH. Advanced maternal age. Part I: obstetric complications. American journal of perinatology 2008;**25**(08):521-34.
41. Huang L, Sauve R, Birkett N, et al. Maternal age and risk of stillbirth: a systematic review. Canadian Medical Association Journal 2008;**178**(2):165-72.
42. Carolan M. Maternal age >45 years and maternal and perinatal outcomes: a review of the evidence. Midwifery 2013;**29**(5):479-89.

For peer review only - http://bmjopen.bmj.com/site/about/guidelines.xhtml

43. Bayrampour H, Heaman M. Advanced maternal age and the risk of cesarean birth: a systematic review. Birth 2010;**37**(3):219-26.
44. D'Agostino Jr RB. Propensity score methods for bias reduction in the comparison of a treatment to a non-randomized control group. Statistics in Medicine 1998;**17**(19):2265-81.
45. Cheslack-Postava K, Liu K, Bearman PS. Closely spaced pregnancies are associated with increased odds of autism in California sibling births. Pediatrics 2011;**127**(2):246-53.
46. Riley RD, Lambert PC, Abo-Zaid G. Meta-analysis of individual participant data: rationale, conduct, and reporting. BMJ 2010;**340**.
47. Egger M, Smith GD, Phillips AN. Meta-analysis: principles and procedures. BMJ : British Medical Journal 1997;**315**(7121):1533-37.
48. Higgins JPT, Thompson SG, Deeks JJ, et al. Measuring inconsistency in meta-analyses. BMJ : British Medical Journal 2003;**327**(7414):557-60.
49. Dildy, Jackson, Fowers, et al. Very advanced maternal age: Pregnancy after age 45. Am J Obstet Gynecol 1996;**175**(3, Part 1):668-74.

For peer review only

For peer review only - http://bmjopen.bmj.com/site/about/guidelines.xhtml

Figure 1: Effects of IPI on preterm birth (PTB) and low birth weight (LBW), with and without matching pregnancies to the same women, Western Australia, 1980-2010

Figures produced from statistics reported in Ball et al. 2014 [26]

For peer review only

Figure 2: Distribution of interpregnancy interval following a live birth, by country/state – 1980-2016

For peer review only

Table 1. Significant gaps in knowledge needed to inform recommendations for optimal interpregnancy interval recommendations in high-income countries.

Knowledge gap	Description
What are the IPI effects after eliminating confounding from between-women comparisons?	Few past studies have addressed confounding by matching pregnancies to the same women.[1]
Can IPI effects be observed later in childhood?	No past studies have investigated outcomes beyond the neonatal period, such as hospitalisation in early childhood.
What are the optimal IPIs for which risks are minimised?	Although knowledge of harmful IPIs is important, health is optimised by identification of optimal IPIs for which risks are minimised.[1]
What are the IPI effects for a wide range of outcomes of both the mother and child?	The vast majority of studies to date investigate a single endpoint for a single group (mother or child). IPI recommendations require a wide range of health endpoints for both mother and child.
What are the context-specific IPI recommendations?	Guideline IPIs are needed for sub-populations e.g., for women who attempt labour after caesarean birth to avoid uterine rupture,[29] and for women after stillbirth due to elevated risk of recurrence or faster maternal recovery. For women at advanced maternal age, delaying child birth even further due to IPI recommendations comes at the cost of competing risks of age-related morbidities.[49]

For peer review only

Table 2. Description of cohort and data sources used to identify a cohort of births in four high-income countries.

**Location**	**California, USA**	**Finland**	**Norway**	**New South Wales, Australia**	**Western Australia, Australia**
**Time Period**	1991-2010	1987-2017	1980-2016	1994-2016	1980-2015
**Data source**	Office of Statewide Health Planning and Development (OSHPD)	National Institute for Health and Welfare (THL), Medical Birth Register	Norwegian Institute of Public Health, Medical Birth Registry	NSW Perinatal Data Collection, NSW Ministry of Health	WA Midwives Data Collection, WA Department of Health
**Information available**	Maternal characteristics and health conditions; smoking and BMI (2007-2010); pregnancy and labour conditions, antenatal hospitalizations, information on delivery and birth outcomes; gestational age based on LMP estimate (obstetric estimate available for years 2007-2010)	Maternal characteristics and health conditions; smoking; pregnancy and labour complications; pregnancy history; details of antenatal care; information on delivery and birth outcomes; health of infant at discharge or 7 days	Maternal characteristics and health conditions; pregnancy and labour complications; medication use during pregnancy; birth outcomes; diagnoses of congenital abnormalities; parental occupation and smoking; births following assisted conception	Maternal characteristics and health conditions; smoking; pregnancy and labour complications; details of labour; birth outcomes; congenital anomalies, infant and child health outcomes	Maternal characteristics and health conditions; smoking; pregnancy and labour complications; details of labour; birth outcomes; congenital anomalies, infant and child health outcomes
**Scope of notified births**	All livebirths and stillbirths with Gestational length ≥20 weeks	All livebirths and stillbirths with gestational length ≥22 weeks or birthweight ≥500g	All pregnancies ending after week 12 from 2002 onwards (from week 16 from 1980-2001)	Gestational length ≥20 weeks or birthweight ≥400g	Gestational length ≥20 weeks or birthweight ≥400g
**Linkage methods**	Probabilistic linkage based on maternal descriptors	Deterministic linkage of mother based on Personal ID	Deterministic linkage of mother based on Personal ID	Probabilistic linkage based on maternal descriptors	Probabilistic linkage based on maternal descriptors
**Total births**	10.9 million	1.8 million	2.1 million	2.2 million	1.2 million
**Benchmark neonatal morbidity indicators**	10.5% PTB (LMP estimate)<br>6.4% LBW	5.8% PTB<br>4.3% LBW	5.7% PTB<br>3.4% LBW	5.4% PTB<br>4.4% LBW	5.3% PTB<br>5.3% LBW

For peer review only

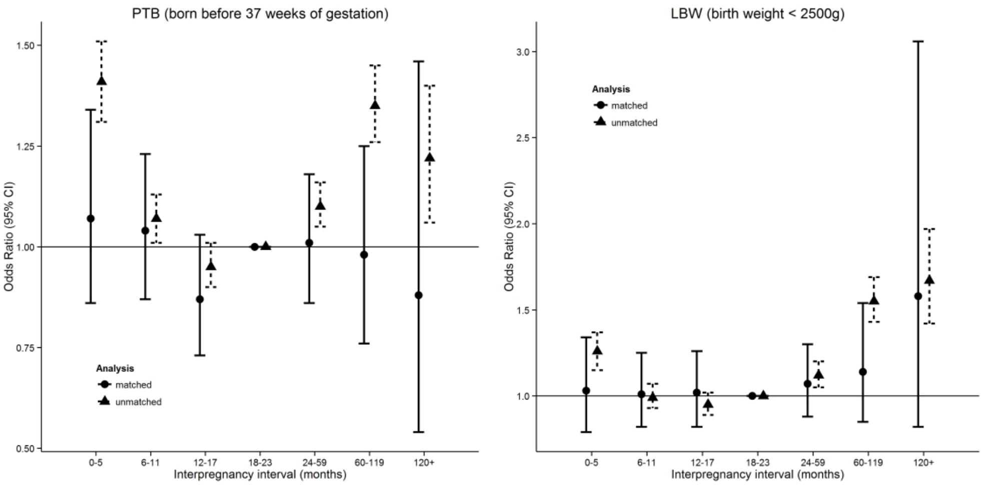

Figure 1: Effects of IPI on preterm birth (PTB) and low birth weight (LBW), with and without matching pregnancies to the same women, Western Australia, 1980-2010

Figures produced from statistics reported in Ball et al. 2014 [26]

355x177mm (300 x 300 DPI)

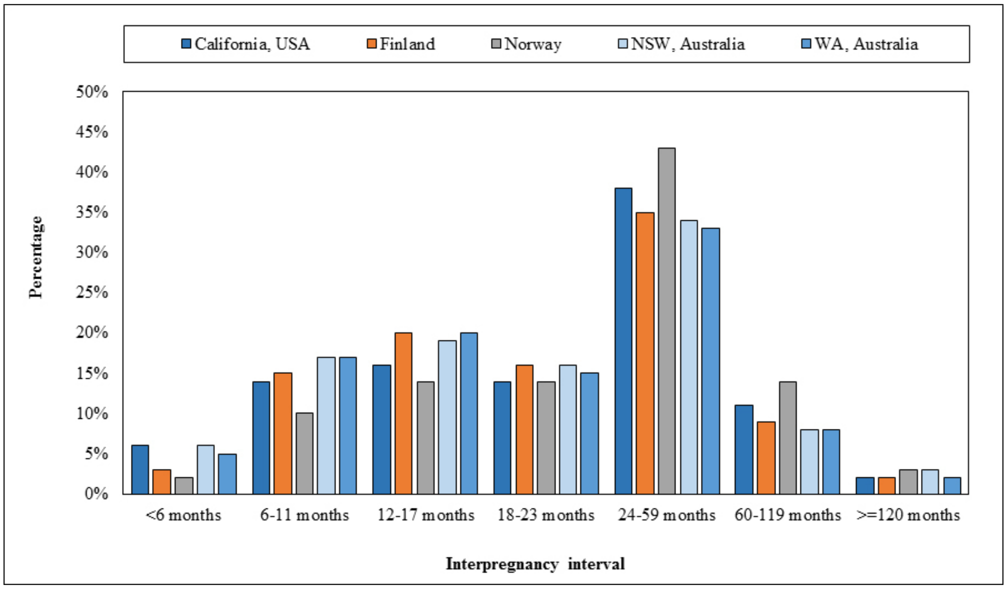

Figure 2: Distribution of interpregnancy interval following a live birth, by country/state – 1980-2016

76x44mm (300 x 300 DPI)

Table S1. Description of outcome ascertainment related to interpregnancy intervals in high-income countries and data sources for measuring outcomes.

Category	Measure/Data source	Description
Preterm birth	Length of gestation (completed weeks)	The quality of gestational length data has improved over time - for all cohorts, frequency of antenatal ultrasounds for pregnancy dating has increased, with corresponding declines in recording gestational lengths based on last menstrual period. PTB will be defined as all births <37 completed weeks gestation, and also sub-classified as idiopathic (spontaneous) and iatrogenic (medically indicated).
Fetal growth restriction based on SGA and low birthweight	Birth weight (grams)	LBW will be defined as weight <2500g. Birth weights are accurately measured by clinical personnel, with near-complete coverage. SGA will be defined as birth weight <10th centile (for gestational length and infant sex) of the birth weight distribution, by calendar year (which accounts for cohort effects), and by cohort (which accounts for differences between countries).
Congenital anomaly	WA, WA Register of Developmental Anomalies (WARDA)<br>NSW, Register of Congenital Conditions (RoCC)<br>Norway, Medical Birth Registry<br>Finland, Register of Congenital Malformations	The WARDA is a comprehensive database of all birth defects diagnosed up to 6 years of age, notifiable by legislation. A 5-year post-birth follow-up period will be used to identify birth defects. For NSW, the RoCC includes all infants diagnosed up to one year of age and only for a five-year period. The comprehensive case identification in WA will enable us to quantify non-identification of cases in the other cohorts, and weight the analyses accordingly.[50] For all cohorts combined, we estimate that >94% of cases with congenital anomaly will be identified.<br>In Finland, data on congenital anomalies are gathered from hospitals, health-care professionals and other national health registers. All cases recorded during pregnancy or infancy (<1 year) are included in the register. Statistical data are used for monitoring congenital anomalies nationally and regionally, and among other purposes, for planning prenatal screening, diagnostics and treatment of congenital anomalies as well as for conducting research on congenital anomalies. https://thl.fi/en/web/thlfi-en/statistics/information-on-statistics/register-descriptions/register-of-congenital-malformations.
Complications	Placental abruption, pPROM,	These databases have coverage of every birth within the state/country, and

For peer review only

of pregnancy and labour	labour dystocia, uterine rupture, pre-eclampsia, gestational hypertension, gestational diabetes<br><br>Midwives Data Collection (WA), Perinatal Data Collection (NSW), maternal antenatal hospitalisations (WA, NSW), birth certificates (California) and the Medical Birth Registers (Finland, Norway).	the complications of labour and pregnancy are recorded at birth. For California, ICD coded complications (ICD-9) of pregnancy and labour that resulted in antenatal hospitalisation are additionally included on the birth certificate records themselves, and will be used to supplement case identification. For WA, NSW and Finland, case identification will be supplemented with a search of the following primary and secondary diagnosis codes (ICD-9/10) of all hospital admissions between start of pregnancy and the discharge date of the birth admission of the mother. Placental abruption: 641.2, O45; labour dystocia: 662.0, O63.0; uterine rupture: 665.1, O71.0, O71.1; pre-eclampsia: 642.4, 642.5, 642.7, O14; gestational hypertension: 642.3, O13; pPROM: 658.1, 658.2 (+ gestational length <37wks), O42; gestational diabetes: 648.8, O24.4
Infant and child health outcomes	Hospital admissions (WA, NSW, Finland)	Hospital contacts will identified from hospital admissions (WA, NSW, Finland) with 3-year follow-up (birth-age 3 years).
Perinatal, infant and maternal mortality	Death registrations (WA, NSW, Norway, Finland) and death certificates (California).	These data sources record every death registered in the state/country. The date of death will be utilised to identify stillbirths (week 20 gestation – birth), perinatal mortality (stillbirths + neonatal mortality), infant mortality (28 days – age 1 year) early childhood mortality (age 1 year to age 3 years), and maternal mortality (pregnancy period – 42 days postpartum). Accidental death, homicide, and other external causes will be identified with cause of death codes and subsequently excluded. Hospital contacts will identified from hospital admissions (WA, NSW, Finland) with 3-year follow-up (birth-age 3 years) and investigated as binary outcomes (>0; >10th,25th,50thcentiles of frequency).

For peer review only

Table S2: Factors affecting pregnancy intervals identified by our Healthy Pregnancy Consumer Reference Group.

Psychological factors	*Replace* a pregnancy loss; compensate for a previous adverse birth outcome; psychological preparation – fear of pregnancy loss or adverse birth outcome in the previous pregnancy; sexual activity is a reminder of pregnancy loss, disability or other adverse birth outcome experienced; relationship stability – partner unsupportive of increasing family size, relationship difficulties, no partner; postnatal depression after the first birth; depression, anxiety, stress, flare of an existing mental health problem or difficulty managing medication while pregnant; experience of a stress-event (death of a loved one, moving home, job loss, divorce/separation); reproductive coercion/abuse.
**Healthcare provision**	Lack of information from healthcare professionals as to clinical recommendations on birth spacing, and their associated risks; encouragement by obstetricians to try again for a child soon after pregnancy loss; information GPs that breastfeeding prevents pregnancy without provision of guidance; no information provided by specialists on risks associated with timing of pregnancy.
**Lifestyle factors/choices**	*Not miss out* - experience what they had planned but could not experience in the first pregnancy; advanced parental age; sex balance desired in family – trying for a girl/boy; household preparation – residential mobility, physical space in the home; ability to manage the day-to-day care of the current child or children – with/without first child morbidities; maternal career aspirations; physical preparation to improve fitness and health; desire to replicate/avoid age gaps in family members; change in partner; multiple gestation in first pregnancy.
**Support and social network**	Availability of support from family – childcare; living in a region distant from extended family; couple living/working in different regions from each other; increase in family size of friends and family; interference from parents/in-laws in the marriage or parenting.
**Contraception sexual activity Fecundity**	Lack of contraceptive use or proper contraceptive use; lack of knowledge regarding pregnancy planning e.g., breast feeding required lactational amenorrhea; failed contraception; higher levels of sexual activity; couple living/working away or work schedules clashing (e.g., shift work); difficulty falling pregnant; reproductive treatment; failed treatment; delays in treatment.
**Financial factors**	Higher paternal income; lower paternal income or reliance on maternal income; cost of contraception; financial preparation – savings, debt; cost of fertility treatment; cost of childcare – more than one child in childcare at the same time is financially prohibitive; paid maternity leave requirements e.g., returning to work for at least 1 year to become eligible.
**Morbidity Healing**	Major morbidities including heart disease, malignant neoplasm; known recurrent pregnancy issues (e.g., hyperemesis gravidarum); healing time required after illness or the previous pregnancy; fear of effects of medication on the fetus; hormonal therapy (progesterone, HCG).

For peer review only
