# Supplementary material for: Developing evidence-based recommendations for optimal interpregnancy intervals in high-income countries: protocol for an international cohort study
Source: BMJ Open. 2019 Jan 29;9(1):e027941. doi: 10.1136/bmjopen-2018-027941 (PMC6352763; doi:10.1136/bmjopen-2018-027941)
Supplement: Reviewer comments [file bmjopen-2018-027941.reviewer_comments.pdf]

PEER REVIEW HISTORY

BMJ Open publishes all reviews undertaken for accepted manuscripts. Reviewers are asked to complete a checklist review form (**http://bmjopen.bmj.com/site/about/resources/checklist.pdf**) and are provided with free text boxes to elaborate on their assessment. These free text comments are reproduced below.

ARTICLE DETAILS

**TITLE (PROVISIONAL)**	Developing evidence-based recommendations for optimal interpregnancy intervals in high-income countries: protocol for an international cohort study
**AUTHORS**	Marinovich, M Luke; Regan, Annette K; Gissler, Mika; Magnus, Maria C; Håberg, Siri Eldevik; Padula, Amy; Mayo, Jonathan; Shaw, Gary M.; Ball, Stephen; malacova, eva; Gebremedhin, Amanuel T; Nassar, Natasha; Marston, Cicely; de Klerk, Nick; Betran, Ana Pilar; Pereira, G.F

This article was not externally reviewed at BMJ Open. Protocols that have been independently assessed prior to submission to BMJ Open are usually fast-tracked to publication on the grounds that further substantial changes will not be possible. This independent assessment will usually be external, independent review for both a substantial grant award from a non-commercial or government funder and ethics approval.
